# Projection of Target Drug Particle Size in Oral Formulations Using the Refined Developability Classification System (rDCS)

**DOI:** 10.3390/pharmaceutics15071909

**Published:** 2023-07-08

**Authors:** Kristian Beran, Eline Hermans, René Holm, Kia Sepassi, Jennifer Dressman

**Affiliations:** 1 Fraunhofer Institute of Translational Medicine and Pharmacology, 60596 Frankfurt am Main, Germany; 2 Janssen Research & Development, Pharmaceutical & Material Sciences, 2340 Beerse, Belgium; 3 Department of Physics, Chemistry and Pharmacy, University of Southern Denmark, 5230 Odense, Denmark; 4 Janssen Research & Development, Discovery Pharmaceutics, La Jolla, CA 92121, USA

**Keywords:** oral drug absorption, particle size, refined developability classification system (rDCS), dissolution limitations, transit, permeability

## Abstract

Dissolution limitations to oral absorption can occur if the time required for dissolution is longer than the transit time across the small intestine and/or if dissolution is slower than the drug’s permeation through the gut wall. These limitations most often occur for poorly soluble drugs. A standard method for overcoming dissolution issues is to reduce the particle size of the (solid) drug. Building on the refined Developability Classification System (rDCS), this work establishes a novel set of equations with which the appropriate degree of particle size reduction needed to mitigate dissolution limitations to absorption can be calculated. According to the type of data available, the appropriate equation(s) for each situation can be applied. Three case examples are used to illustrate implementation of the equations: voriconazole, lemborexant and istradefylline. Although for voriconazole (rDCS Class I) target radius (r_target_) estimates indicate that particle size reduction is unnecessary, for lemborexant (rDCS Class I) a radius of ≤20 µm would be required to improve absorption. For istradefylline (rDCS Class IIb) the r_target_ was approximately 12 µm. Results are commensurate with literature information for these three drugs, signaling that the equations are suitable for application to a wide variety of drug substances.

## 1. Introduction

The dynamic interplay between dissolution rate, solubility and permeability in the gastrointestinal tract (GIT) is fundamental to understanding oral drug absorption and serves as the basis for the Biopharmaceutics Classification System (BCS) [1]. Orally administered active pharmaceutical ingredients (APIs) must first dissolve in the GI fluids and be absorbed through the gut wall to enter the systemic circulation and subsequently reach their pharmacological target. Dissolution rate limitations to oral drug absorption can occur if the time required for dissolution of an API is longer than the transit time across the absorptive sites in the GIT and/or if dissolution in the GI fluids is significantly slower than the drug’s permeation through the intestinal epithelium [2,3]. The dissolution process of solids applying a diffusion layer model was first described by Noyes and Whitney in 1897 and modified by Nernst and Brunner in the early 1900s [4,5,6,7]. The Noyes–Whitney/Nernst–Brunner equation describes the mass transfer rate from the dissolving surface into the fluid, which is dependent on the surface area exposed to the dissolution medium, the hydrodynamic conditions, the diffusion coefficient, and the thermodynamic solubility of the dissolving molecule. Due to the reciprocal relationship between particle radius and total powder surface area, dissolution rates can be increased through particle size reduction. In cases where dissolution is slower than permeation, the particle size distribution can have a significant impact on the bioavailability of APIs formulated as solid or suspension formulations, necessitating appropriate particle size specifications to ensure efficacious, safe, and reproducible therapy [8]. 

The Developability Classification System (DCS) and refined Developability Classification System (rDCS) are tools for assessing the developability of new APIs, including the probability of dissolution rate limitations and target particle size [9,10,11]. 

The DCS was introduced in 2010 and has since found widespread application in both the pharmaceutical industry and academia to predict biopharmaceutical challenges and limitations to oral drug absorption. As an adaption of the BCS, the DCS was conceived specifically to address the demands of drug development and guide formulation strategies to guarantee adequate in vivo drug exposures in (pre-) clinical studies. The DCS classifies APIs into five classes according to the key drivers for absorption: dose, intestinal solubility, and permeability [9]. High small intestinal solubility and permeability result in a low risk of incomplete absorption for APIs in class I, such as paracetamol, whereas permeability limitations can be expected for APIs in class III, such as low-dose acyclovir [9]. Based on the concept that solubility and permeability are to a certain degree compensatory, the DCS introduced the solubility-limited absorbable dose (SLAD), thereby splitting DCS class II into IIa and IIb for dissolution rate limited APIs (e.g., mefenamic acid) and solubility-limited APIs (e.g., griseofulvin), respectively [9]. APIs belonging to class IV, such as high-dose acyclovir, present challenges in formulation development due to both limited solubility and permeability [9]. In addition to the revised classification procedure, another innovation of the DCS was the implementation of a calculated target particle size (“r_target_”) based on the Dissolution number (Dn), below which dissolution rate limitations can be mitigated [9].

To provide a toolbox specifically tailored to the early stages in oral drug development, the DCS was further expanded into the rDCS. An investigational decision tree with standard and customized investigations to better account for the heterogeneity of APIs in the development pipeline was introduced [10,11]. The rDCS standard investigations are based on the classical DCS but incorporate the following modifications [10,11]:

(i) The calculation of the dose-to-solubility (D/S) ratio utilizes a standard dose range (5, 50, and 500 mg) instead of a fixed dose, since in preclinical development the human therapeutic dose has typically not yet been defined.
(ii) The solubility in fasted state human intestinal fluid (FaHIF) is estimated through a correlation between in vitro solubilities in in-house biopredictive media.
(iii) The effective human permeability (P_eff_) is estimated by establishing a correlation between in-house permeability data and reported permeabilities in human volunteers.

The standard investigations indicate whether the risk associated with the development of the API as an immediate release (IR) formulation will be high, medium or low. Depending on the risk analysis and the API’s physicochemical properties, further customized investigations may be necessary to fully capture the developability risk and make meaningful recommendations about the formulation strategy selection. Customized investigations include small-scale biorelevant supersaturation/precipitation assays for weak bases or salts of weak acids that are classified as rDCS class IIb or IV. For APIs classified as rDCS class IIa or class I/III, but with an aqueous solubility below 100 µg/mL, more extensive dissolution rate analysis, involving small scale intrinsic dissolution rate (IDR) experiments, is recommended. Following the rDCS strategy, the dissolution rate constant (k_diss_) is compared with the permeation rate constant (k_perm_) to estimate whether dissolution or permeation will be rate-limiting to oral absorption [10,11].

In this work we explored and compared different ways of estimating the API particle size, r_target_, below which its absorption from an immediate release solid oral dosage form will not be impeded by a low dissolution rate. Building on the DCS and rDCS approaches to assess dissolution rate limitations, a new set of analytically solvable equations for the estimation of r_target_ was developed. Results for three APIs (voriconazole, lemborexant and istradefylline) covering a broad range of solubility in fasted state simulated intestinal fluid (FaSSIF V1) were compared. 

## 2. Theoretical Section

In this section, various approaches to calculation of r_target_ are shown. Detailed derivations of the equations presented can be found in Supplementary Materials.

### 2.1. Dissolution/Transit Balance: Target Particle Size Estimations Based on the Dissolution Number and Solubility 

The Dissolution Number, Dn, is a dimensionless number developed in a microscopic mass balance approach to estimate the fraction of dose absorbed from suspensions and assess the probability of dissolution rate limitations to oral absorption [12]. The value is an integral part of the BCS and DCS and represents the relative rate of dissolution to axial convection, expressed as the ratio of intestinal residence time (Equation (1)) to dissolution time (Equation (2)) from particles with a defined initial particle radius r_0_ [1,12]. The Dn can be calculated from Equations (3) and (4):

(1) $$T_{si}=\frac{\pi {\cdot R_{si}}^{2}\cdot L_{si}}{Q}$$

(2) $$T_{diss}=\frac{1}{k_{diss}}=\frac{r_{0}\cdot h_{particle}\cdot \rho }{3\cdot D\cdot C_{s}}$$

(3) $$Dn=\frac{T_{si}}{T_{diss}}=\frac{3\cdot D}{r_{0}\cdot h_{particle}}\cdot \frac{C_{s}}{\rho }\cdot T_{si}$$

(4) $$Dn=k_{diss}\cdot T_{si}$$

where T_si_ is the mean small intestinal transit time, R_si_ is the small intestinal radius, L_si_ is the small intestinal length, Q is the intestinal fluid flow rate, T_diss_ is the time for a particle to dissolve, k_diss_ is the dissolution rate constant, r_0_ is the initial particle radius, h_particle_ is the thickness of the aqueous boundary layer (ABL), ρ is the API density, D is the diffusion coefficient and C_S_ is the thermodynamic solubility [1,2,3,12].

T_si_ is a physiological parameter mainly determined by GI motility and its underlying intra- and interindividual variability. For the calculations in this work, T_si_ was set to 3.32 h, in line with the DCS and rDCS calculations [9,10]. In certain circumstances T_si_ may need to be adjusted, e.g., if it is known that an excipient or co-administered API affects T_si_ [13,14,15], in certain disease states, or if the drug has an “absorption window”. 

Since the dissolution rate, and hence T_diss_, can be modulated through particle size adjustment, they are important variables in the development of oral dosage forms. The reciprocal of T_diss_ is referred to as k_diss_ (Equations (5) and (6)) [2,3], which can be derived by rearrangement of the Noyes–Whitney/Nernst–Brunner equation under key assumptions regarding the total powder surface area (A_powder_), sink conditions, and the thickness of the ABL of the dissolving particle (h_particle_), as set out in Table 1. 

In the literature, h_particle_ has been assumed to be equal to the particle radius for small particles [17]. As the particle size increases, h_particle_ tends to approach a constant value that is small relative to r_0_ [18,19,21]. For particles with r_0_ < 30 µm, h_particle_ is often assumed to equal the particle radius, whereas for r_0_ ≥ 30 µm cases, h_particle_ is often assumed to be constant and set to, e.g., 30 µm [3,18,19,20]. This 30 µm cut-off was approximated by calculating the ABL thickness of a rotating disk of compressed API [18]. Depending on r_0_, the h_particle_ value can be adjusted in the k_diss_ equation as shown in Equations (5) and (6) [3].

(5) $$k_{diss}=\frac{3\cdot D}{{r_{0}}^{2}}\cdot \frac{C_{S}}{\rho }\;\left(r_{0}<30\;µm,\;h_{\text{particle}}=r_{0}\right)$$

(6) $$k_{diss}=\frac{3\cdot D}{r_{0}\cdot h_{particle}}\cdot \frac{C_{S}}{\rho }\;\left(r_{0}\geq 30\;µm,\;h_{\text{particle}}=30\;µm\right)$$

Based on the relationship between particle size, dissolution rate and solubility implied by the Dn, Butler and Dressman introduced Equation (7) for the calculation of r_target_ [9]. In the following, this approach is referred to as the “dissolution/transit balance approach”, which answers the question: How low does r_target_ need to be for complete dissolution of the dose within the intestinal transit time under sink conditions? A target Dn of 1 was set, corresponding to complete dissolution of the dose within the small intestinal transit time (assuming T_si_ = 3.32 h) under sink conditions. The r_target_ values calculated from the Dn are especially relevant for APIs in rDCS class IIa and may also be relevant for low solubility (<100 µg/mL) APIs in class I and III, for which a low dose can lead to a D/S ratio of <500 mL. Equation (8) is a modified version of Equation (7) which fixes h_particle_ = 30 µm when the particle size exceeds 30 µm. Both equations can be applied to a given drug, subsequently identifying the most relevant r_target_ value from the results.

(7) $$r_{target}=\sqrt{\frac{3\cdot D\cdot C_{s}\cdot T_{si}}{{Dn}_{target}\cdot \rho }}\;\left(r_{0}<30\;µm,\;h_{\text{particle}}=r_{0}\right)$$

(8) $$r_{target}=\frac{3\cdot D\cdot C_{s}\cdot T_{si}}{{Dn}_{target}\cdot \rho \cdot h_{particle}}\;\left(r_{0}\geq 30\;µm,\;h_{\text{particle}}=30\;µm\right)$$

### 2.2. Dissolution/Transit Balance: Target Particle Size Estimates Based on the Dissolution Number and Intrinsic Dissolution Rate 

Rosenberger et al. published an equation for k_diss_ based on the intrinsic dissolution rate (IDR) rather than C_S_ (Equation (9)) [10,11]. Applying the h_particle_ assumption for larger particles with r_0_ ≥ 30 µm (h_particle_ = 30 µm) results in Equation (10).

(9) $$k_{diss}=k_{IDR}\cdot IDR=\frac{3\cdot h_{disk}}{{r_{0}}^{2}\cdot \rho }\cdot IDR\;\left(r_{0}<30\;µm,\;h_{\text{particle}}=r_{0}\right)$$

(10) $$k_{diss}=k_{IDR}\cdot IDR=\frac{3\cdot h_{disk}}{r_{0}\cdot h_{particle}\cdot \rho }\cdot IDR\;\left(r_{0}\geq 30\;µm,\;h_{\text{particle}}=30\;µm\right)$$

In these equations k_IDR_ is the IDR rate constant, which accounts for the solvent kinematic viscosity, rotational speed of the compressed disk, diffusion coefficient, particle size and API density [10,11]. Therefore, k_IDR_ corrects for differences in experimental conditions that influence the measured IDR value. The ABL thickness of the rotating disk (h_disk_) can be estimated according to Equation (11):

(11) $$h_{disk}=4.98\cdot \nu^{\frac{1}{6}}\cdot D^{\frac{1}{3}}\cdot {RPM}^{-\frac{1}{2}}$$

where ν is the solvent kinematic viscosity and the stirring rate is expressed as revolutions per minute (RPM) [22,23]. When k_diss_ is based on the IDR, two modified Dn equations result (Equations (12) and (13)):

(12) $$Dn=\frac{3\cdot h_{disk}}{{r_{0}}^{2}\cdot \rho }\cdot IDR\cdot T_{si}\;\left(r_{0}<30\;µm,\;h_{\text{particle}}=r_{0}\right)$$

(13) $$Dn=\frac{3\cdot h_{disk}}{r_{0}\cdot h_{particle}\cdot \rho }\cdot IDR\cdot T_{si}\;\left(r_{0}\geq 30\;µm,\;h_{\text{particle}}=30\;µm\right)$$

Through rearrangement of Equations (12) and (13), new r_target_ equations based on Dn and IDR can be obtained (Equations (14) and (15)).

(14) $$r_{target}=\sqrt{\frac{3\cdot h_{disk}\cdot IDR\cdot T_{si}}{{Dn}_{target}\cdot \rho }}\;\left(r_{0}<30\;µm,\;h_{\text{particle}}=r_{0}\right)$$

(15) $$r_{target}=\frac{3\cdot h_{disk}\cdot IDR\cdot T_{si}}{{Dn}_{target}\cdot \rho \cdot h_{particle}}\;\left(r_{0}\geq 30\;µm,\;h_{\text{particle}}=30\;µm\right)$$

### 2.3. Dissolution/Permeation Balance: Target Particle Size Estimates Based on k_diss_/k_perm_ and Solubility 

The dissolution rate analysis can be extended by a novel approach equating the maximum dissolution rate and maximum permeation rate (Equation (16)) [3]. The maximum dissolution rate term (based on the assumptions listed in Table 1) is defined by k_diss_ and the dose, while the maximum permeation rate is driven by the maximum luminal concentration (expressed as the amount of drug that can be dissolved in the available volume of intestinal fluid, m_dissolved_) and its permeation rate constant (k_perm_) [3]. For most rDCS class I and III drugs, m_dissolved_ equals the dose because the drug is expected to dissolve completely in the intestinal fluid. For rDCS class II and IV drugs, the solubility rather than the dose represents the limiting factor, such that m_dissolved_ will equal the solubility multiplied by the intestinal fluid volume (assumed to be effectively 500 mL). 

The k_perm_ equation (Equation (17)) can be derived from the Absorption Number (An, Equation (30)), which represents the ratio of the radial absorption rate to axial convection rate [12]. Equations (18) and (19) are obtained by integrating the k_diss_ equations for r < 30 µm and r ≥ 30 μm cases (Equations (5) and (6)) as well as the k_perm_ equation (Equation (17)) into Equation (16).

(16) $$k_{diss}\cdot Dose=k_{perm}\cdot m_{dissolved}$$

(17) $$k_{perm}=\frac{{DF\cdot P}_{eff}}{R_{si}}$$

(18) $$\frac{3\cdot D}{{r_{0}}^{2}}\cdot \frac{C_{S}}{\rho }\cdot Dose=\frac{{DF\cdot P}_{eff}}{R_{si}}\cdot m_{dissolved}\;\left(r_{0}<30\;µm,\;h_{\text{particle}}=r_{0}\right)$$

(19) $$\frac{3\cdot D}{r_{0}\cdot h_{particle}}\cdot \frac{C_{S}}{\rho }\cdot Dose=\frac{{DF\cdot P}_{eff}}{R_{si}}\cdot m_{dissolved}\;\left(r_{0}\geq 30\;µm,\;h_{\text{particle}}=30\;µm\right)$$

In addition to the variables that have been defined earlier, DF is the degree of flatness of the intestine and P_eff_ is the estimated human effective small intestinal permeability. 

By rearranging Equations (18) and (19) for r_0_, new r_target_ equations (Equations (20) and (21)) can be derived, which will subsequently be referred to as the “dissolution/permeation balance approach”. This approach provides an answer to the question: How low does r_target_ need to be for permeation rather than dissolution to become rate limiting?

(20) $$r_{target}=\sqrt{\frac{3\cdot D\cdot Dose{\cdot R}_{si}{\cdot C}_{s}}{DF\cdot P_{eff}\cdot \rho {\cdot m}_{dissolved}}}\;\left(r_{0}<30\;µm,\;h_{\text{particle}}=r_{0}\right)$$

(21) $$r_{target}=\frac{3\cdot D\cdot Dose{\cdot R}_{si}{\cdot C}_{s}}{DF\cdot P_{eff}\cdot \rho {\cdot m}_{dissolved}\cdot h_{particle}}\;\left(r_{0}\geq 30\;µm,\;h_{\text{particle}}=30\;µm\right)$$

### 2.4. Dissolution/Permeation Balance: Target Particle Size Estimates Based on k_diss_/k_perm_ and Intrinsic Dissolution Rate 

An alternative approach is to apply the k_diss_ based on the IDR (Equations (9) and (10)) to estimate the maximal dissolution rate in Equation (16). Through rearrangement of these equations, further r_target_ equations based on the dissolution/permeation balance and the IDR as input can be derived (Equations (22) and (23)):

(22) $$r_{target}=\sqrt{\frac{3\cdot h_{disk}\cdot Dose{\cdot R}_{si}\cdot IDR}{DF\cdot P_{eff}\cdot \rho {\cdot m}_{dissolved}}}\;\left(r_{0}<30\;µm,\;h_{\text{particle}}=r_{0}\right)$$

(23) $$r_{target}=\frac{3\cdot h_{disk}\cdot Dose{\cdot R}_{si}\cdot IDR}{DF\cdot P_{eff}\cdot \rho {\cdot m}_{dissolved}\cdot h_{particle}}\;\left(r_{0}\geq 30\;µm,\;h_{\text{particle}}=30\;µm\right)$$

### 2.5. Dissolution/Permeation Balance: Target Particle Size Estimates Based on Powder Surface Area and Intrinsic Dissolution Rate 

The dissolution rate from a rotating disk of compressed API can be calculated by multiplying the IDR by the constant disk surface area (A_disk_). This approach can also be applied to the dissolution of a powder, whereby the powder dissolution rate can be estimated from the IDR and the total powder surface area (A_powder_) according to Equation (24):

(24) $$\frac{dM}{dt}=IDR\cdot A_{powder}$$

This definition of the dissolution rate can be related to the maximum permeation rate analogous to Equation (16). Integration of the A_powder_ equation presented in Table 1 yields Equation (25), and rearrangement results in another r_target_ equation (Equation (26)).

(25) $$IDR\cdot \frac{3\cdot Dose}{r_{0}\cdot \rho }=\frac{{DF\cdot P}_{eff}}{R_{si}}\cdot m_{dissolved}$$

(26) $$r_{target}=\frac{3\cdot Dose{\cdot R}_{si}\cdot IDR}{DF\cdot P_{eff}\cdot \rho {\cdot m}_{dissolved}}\;\left(h_{\text{particle}}=h_{\text{disk}}\right)$$

The dissolution rate term using A_powder_ assumes that the disk IDR is a constant which can be projected to powder dissolution without corrections. Thus, the approach assumes that h_particle_ during powder dissolution equals h_disk_, noting that h_disk_ values under common experimental conditions (100 rpm) often exceed the 30 µm cut-off [22].

### 2.6. Summary of Target Particle Size Equations

**Figure 1:**
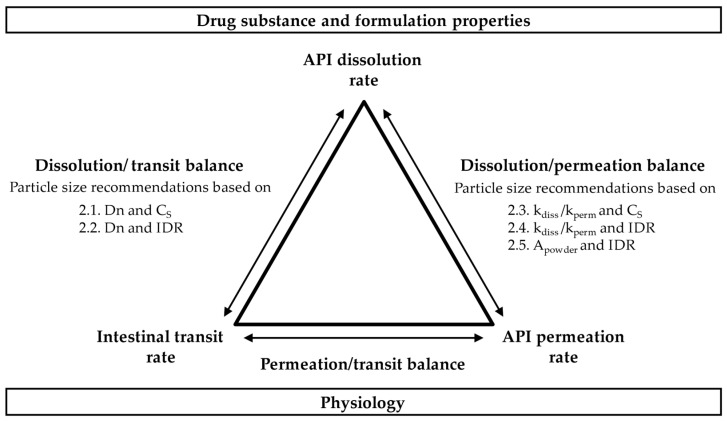
Visualization of the theory behind the r_target_ equations.

In this analysis, nine key equations, including eight new ones, were derived for the calculation of the target particle size in early drug development. The theory behind the approaches presented in Section 2.1, Section 2.2, Section 2.3, Section 2.4 and Section 2.5 is visualized in Figure 1. 

The equations, summarized in Table 2, are based either on C_S_ or IDR as input parameters, include different assumptions about the ABL thickness and were constructed to examine the dissolution/transit and dissolution/permeation balance in various scenarios, as shown in Figure 1. To facilitate implementation of the equations, an Excel spreadsheet for automated calculation of all r_target_ values is provided as Supplementary Materials. The permeation/transit balance is not interrogated in this work, as neither is influenced directly by particle size.

**Figure 2:**
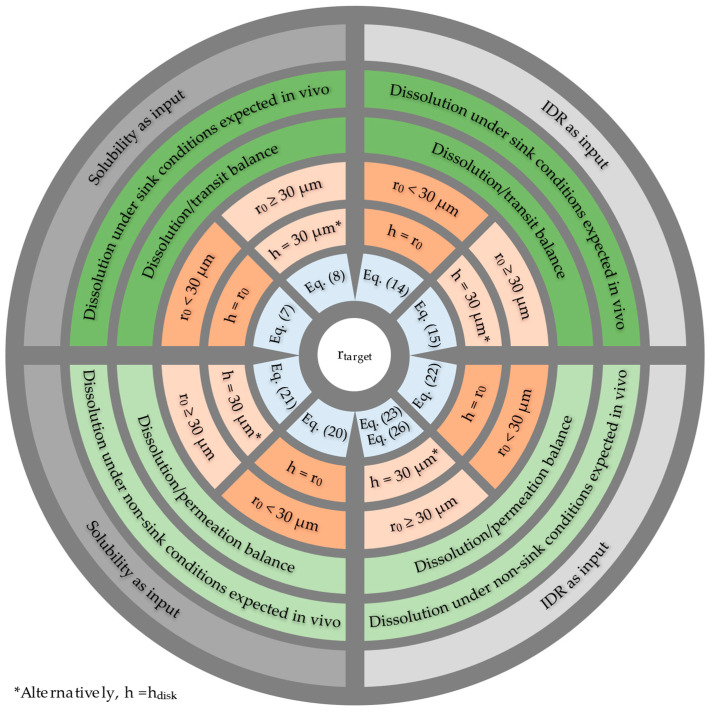
Decision wheel for selection of the appropriate r_target_ equation(s) for a given case. The figure should be read from the outermost shell inwards. Detailed equations are presented in Table 2. Further details are provided in the above text.

Figure 2 provides guidance on which equation may be best suited for a given case, noting that it may be prudent to compare r_target_ values calculated using different approaches to capture the most relevant particle size. Starting from the outermost shell, the first step involves selecting the preferred input parameter for the r_target_ calculations, which can be either solubility (C_S_) or intrinsic dissolution rate (IDR). Depending on the availability of experimental data, particularly in cases where the rDCS recommends customized IDR experiments, values calculated from C_S_ and IDR should be compared to understand whether factors like wetting of the API surface or reactions in the ABL influence the dissolution kinetics. 

The second step involves estimating whether in vivo dissolution will take place under sink conditions. If sink conditions are expected to apply, the dissolution/transit balance approach should be used and, alternatively, if sink conditions are not expected to be met, the dissolution/permeation balance approach is recommended. Sink conditions are likely to be maintained if the permeability of the API is high and/or if the D/S ratio is low such that concentrations in the intestinal fluid are kept well below one-third of the saturation concentration. Conversely, sink conditions are less likely to apply if the permeability is medium to low and/or if the D/S ratio is high. In the innermost shells, a suitable assumption regarding the ABL thickness is chosen. If the calculated r_target_ is <30 µm, the assumption h_particle_ = r_0_ is an appropriate option. On the other hand, if the calculated r_target_ is ≥30 µm, the assumptions h_particle_ = 30 µm or h_particle_ = h_disk_ are appropriate. At this point, the recommended r_target_ equation(s) is selected.

## 3. Materials and Methods

### 3.1. Chemicals 

**Figure 3:**
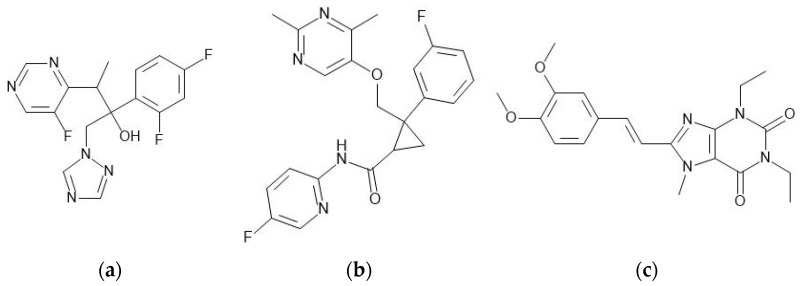
Chemical structures of (**a**) voriconazole, (**b**) lemborexant and (**c**) istradefylline (drawn with MedChem Designer™ version 5, Simulations Plus Inc., Lancaster, CA, USA).

Voriconazole (batch FV291011901, purity > 99%) and lemborexant (batch 0000041758, purity > 99%) were purchased from Biosynth Carbosynth (Compton, UK). Istradefylline (batch 16512, purity > 99%) was purchased from MedChemExpress LLC (Monmouth Junction, NJ, USA). The chemical structures of the three APIs are shown in Figure 3.

Acetonitrile (ACN gradient grade for liquid chromatography), dimethylsulfoxide (DMSO for liquid chromatography), hydrochloric acid (5 N HCl) and sodium hydroxide (5 N NaOH) were purchased from Merck KGaA (Darmstadt, Germany). Trifluoroacetic acid (TFA) was purchased from Thermo Fisher Scientific (Rockford, IL, USA). FaSSIF/FeSSIF/FaSSGF powder and FaSSIF buffer concentrate were purchased from Biorelevant.com Ltd. (London, UK). Water was purified with a Milli-Q Advantage A10 Water Purification System (Merck KGaA, Darmstadt, Germany). 

FaSSIF V1 was prepared according to the instructions provided by Biorelevant.com [24]. First, a FaSSIF blank buffer was prepared by diluting the buffer concentrate with Milli-Q water. The final medium was obtained by dissolving FaSSIF powder in the blank buffer and equilibrating the medium at room temperature for 2 h. The pH was measured and adjusted to pH 6.50 ± 0.05 using hydrochloric acid or sodium hydroxide, if necessary.

### 3.2. Physicochemical Characterization

#### 3.2.1. Dissociation Constant (pK_a_)

The pK_a_ values of voriconazole, lemborexant and istradefylline were determined at Pion Inc (UK) Ltd. (Forest Row, UK). A high-throughput spectrometric screening assay (Fast UV method) using the SiriusT3 (Pion Inc., Billerica, MA, USA) was applied. Samples were titrated under varying water/methanol ratios and apparent pK_a_ values were extrapolated using the Yasuda–Shedlovsky extrapolation. The experimental details are provided in Supplementary Materials. Measured pK_a_ values are presented as means (±SD) and were compared to literature values as well as in silico predictions using the software ADMET Predictor version 9 (Simulations Plus Inc., Lancaster, CA, USA) and ACD/Labs Release 2021.1.2 (Advanced Chemistry Development Inc., Toronto, ON, Canada).

#### 3.2.2. n-Octanol/Water Distribution Coefficient (logD_pH7.4_)

LogD_pH7.4_ values of the three APIs were determined at Pion Inc (UK) Ltd. (Forest Row, UK) using a shake-flask screening method and high performance liquid chromatography with UV detection (HPLC-UV). Samples containing the API were equilibrated between octanol-saturated 0.01 M phosphate buffer at pH 7.4 and buffer-saturated octanol and analyzed by HPLC-UV. The experimental details are provided in Supplementary Materials. Measured logD_pH7.4_ values are presented as means (±SD) and were compared to literature values as well as in silico predictions using the software ADMET Predictor version 9 and ACD/Labs Release 2021.1.2.

### 3.3. rDCS Standard Investigations: Solubility Studies

Equilibrium solubilities of the three APIs in FaSSIF V1 were measured with the shake-flask method, following the ICH M9 guideline on BCS-based biowaivers [15]. Briefly, an excess amount of each API was weighed into a 5 mL vial and the assay medium (FaSSIF V1) was added. The resulting suspensions were incubated at 37.0 ± 0.5 °C (Memmert Incubator IN750, Memmert GmbH + Co. KG, Schwabach, Germany) and agitated for 24 h at 25 rpm (LABINCO LD76 Digital Rotary Mixer, Labinco BV, Breda, The Netherlands). At 2, 4, 6 and 24 h, samples were drawn and filtered through Acrodisc 13 mm with 0.45 µm PTFE filters (Pall Corporation, Port Washington, NY, USA) after establishing that adsorption of the APIs onto the filters was less than 5%. Before sampling, the suspensions were checked visually for the presence of solid particles and the pH was measured (Metrohm 780 pH Meter, Metrohm AG, Herisau, Switzerland). All measured pH values were within the specified range of pH 6.50 ± 0.05, so no pH adjustment was necessary. The filtrates were diluted with a 1:1 mixture of ACN and Milli-Q water for analysis using ultra-performance liquid chromatography with UV detection (UPLC-UV). 

Residual solids from the solubility studies were collected after 24 h and examined using X-ray powder diffraction (XRPD) analysis. XRPD patterns of the residual solids were compared to the starting material and literature profiles. A differential scanning calorimetry (DSC) analysis of the input materials was additionally performed. Istradefylline samples were protected from light in all studies using amber vials and aluminum foil to prevent photolysis [24]. The experimental details of the UPLC-UV methods and solid state analyses can be found in Supplementary Materials. 

The solubility experiments were performed in triplicate (*n* = 3) and solubilities are presented as means (± SD). The mean values were used as input for the rDCS and r_target_ calculations. 

### 3.4. rDCS Standard Investigations: Caco-2 Permeability Studies

In vitro permeability studies were performed at Cyprotex Discovery Ltd. (Macclesfield, UK) using a high-throughput Caco-2 permeability assay. Permeability at 37 °C was assessed from the apical to basolateral side (A⟹B) and in opposite direction (B⟹A) applying a pH gradient with pH 6.5 in the apical and pH 7.4 in the basolateral compartment (Hanks Balanced Salt Solution, HBSS). After an incubation of 120 min, samples were diluted for analysis via liquid chromatography coupled with tandem mass spectrometry (LC-MS/MS). Measurements were performed in duplicate and reference compounds of known permeability (atenolol, propanolol and antipyrine) were included as quality controls. Experiments were performed in absence and presence of the P-glycoprotein (P-gp) inhibitor elacridar (10 µM) to study transporter-mediated efflux effects. The dosing solutions contained the API (10 µM) in HBSS, and DMSO in a concentration of ≤1% *v*/*v*. Lucifer yellow (100 µM) was included as fluorescent integrity marker. Further experimental details are provided in Supplementary Materials.

The apparent permeability coefficients (P_app_) were calculated according to Equation (27):

(27) $$P_{app}=\left(\frac{dQ/dt}{C_{0}\cdot A}\right)$$

where dQ/dt is the permeation rate of the API across the cell monolayer, C_0_ is the starting concentration of the API in the dosing solution and A is the surface area of the cell monolayer [25]. The efflux ratios (ERs) were calculated from the B⟹A and A⟹B P_app_ values to estimate whether P-gp mediated efflux was involved in the transport of the APIs. The mass balance (% recovery) was calculated from the sum of the API recovered from the acceptor and donor compartments at the end of the experiment, divided by the initial donor amount [25].

The Caco-2 P_app_ values were averaged. P_app_(A⟹B) values without inhibitor were converted to effective human in vivo permeability (P_eff_) values using Simcyp Version 21 software (Simcyp Ltd., Sheffield, UK). The built-in literature P_app_–P_eff_ correlation assuming a pH gradient (pH 6.5⟹7.4) and passive API permeation with atenolol and propranolol as calibrator substances was used [26]. Estimated P_eff_ values were used as input for the rDCS and r_target_ calculations.

### 3.5. rDCS Customized Investigations: Intrinsic Dissolution Rate 

#### 3.5.1. Disk Dissolution Studies

The IDRs of voriconazole and lemborexant in FaSSIF V1 at 37.0 ± 0.5 °C were determined using the µDISS Profiler (Pion Inc., Billerica, MA, USA) [27]. The experimental design was adapted from a standard experimental protocol published previously [28]. Disks (*n* = 3) with a surface area of 0.0707 cm^2^ were prepared by compressing the APIs into disk dies. For this, 5 to 10 mg of the API was compressed for 1 min at 50 bar using a Mini-IDR Tablet Compression System (Heath Scientific Co. Ltd., Milton Keynes, UK). Loose powder on the disk surface or die was removed with compressed air. The disks were prepared from the pure crystalline form of voriconazole (form B) and lemborexant (form CS2). With the disk surface facing upwards, the dies were placed into round-shaped magnetic stirrers and transferred into 20 mL assay vials. The vials were placed in a heating block and pre-heated FaSSIF V1 (10 mL) was added to each vial to start the dissolution experiments. The disks were rotated at 100 rpm and concentrations were measured in situ with UV-spectroscopy using fiber optic dip probes. Experiments were performed in triplicate. 

The dissolution curves were truncated at concentrations corresponding to 10% of the solubility to ensure sink conditions and the IDR was calculated from the slope of the dissolution profile according to Equation (28):

(28) $${IDR}_{disk}=\frac{dC}{dt}\cdot V\cdot \frac{1}{A_{disk}}$$

where dC/dt is the slope of the linear concentration-time curve, t is the time, V is the volume of the dissolution medium and A_disk_ is the disk surface area [22,27,28]. Measured IDRs were compared to theoretical IDRs calculated from the Levich equation with solubility as input via an unpaired t-test with α = 0.05.

XRPD analysis of the disks before and after dissolution was performed, and lemborexant disks were additionally analyzed by DSC. The diffractograms and thermograms can be found in Supplementary Materials.

#### 3.5.2. Powder Dissolution Studies

Istradefylline disks could not be prepared because disk compression led to a change in solid state. The IDR of istradefylline was therefore measured (*n* = 4) according to a published powder dissolution method for estimating rotating disk IDRs [22,28]. Istradefylline was weighed into an assay vial containing a magnetic crossbar stirrer, in an amount corresponding to approximately twice the amount needed to saturate the medium. The vials were placed in a heating block and 20 mL pre-heated FaSSIF V1 (37.0 ± 0.5 °C) was added to start the dissolution experiments. The medium was stirred at 100 rpm and concentrations were measured with in situ UV-spectroscopy using fiber optic dip probes until a plateau in the dissolution profile was reached. The vials were protected from light with aluminum foil during the entire experiment. 

The powder IDR was derived using the dissolution curve tool integrated in the AuPRO Software Version 6 (Pion Inc., Billerica, MA, USA) as described in Tsinman et al. [22]. 

IDRs are presented as means (±SD). Mean values were used as input for the rDCS and r_target_ calculations. 

### 3.6. rDCS Calculations 

#### 3.6.1. rDCS Classification Based on Standard Investigations

rDCS analysis was performed as described by Rosenberger et al. [10,11]. The D/S ratios for voriconazole, lemborexant and istradefylline were calculated based on their biorelevant solubility in FaSSIF V1 and the rDCS standard dose range (5, 50 and 500 mg) [10]. Additional D/S ratios for usual single doses were also calculated: 200 mg for voriconazole [29], 10 mg for lemborexant [30] and 20 mg for istradefylline [31]. The SLAD was calculated according to Equation (29):

(29) $$SLAD=C_{S}\cdot V\cdot M_{p}$$

where C_S_ is the measured thermodynamic solubility, V is the assumed effective volume of intestinal fluids (500 mL), and M_p_ is the permeability dependent multiplier, which is equal to the An for rDCS Class II drugs [9,10]. The An can be calculated according to Equation (30):

(30) $$An=\frac{DF\cdot P_{eff}}{R_{si}}\cdot T_{si}$$

The rDCS classification according to the standard investigations was based on the D/S ratios, P_eff_ and SLAD.

#### 3.6.2. rDCS Customized Investigations: Dissolution Rate Analysis 

The rDCS dissolution rate analysis was conducted according to Rosenberger et al. [10,11]. For this, k_diss_ based on the IDR for a standard particle size of 100 µm was calculated (Equation (15)) and compared to k_perm_ (Equation (10)). The kinematic viscosity of the medium (0.00696 cm^2^/s), aqueous diffusion coefficient (predicted with ADMET Predictor version 9), stirring rate (100 rpm), initial particle size (100 µm), API density (predicted with ACD/Labs Release 2021.1.2), and measured IDR were used to calculate k_diss_. Predicted diffusion coefficients and API densities can be found in Supplementary Materials. The estimated P_eff_, degree of flatness (1.7) and small intestinal radius (2 cm) were used for the calculation of k_perm_. 

As an extension of the original rDCS analysis, the equations presented in the Theoretical Section were used to estimate the r_target_ values for the three APIs. Additional input values for the r_target_ calculations were the measured thermodynamic solubility in FaSSIF V1, the mean small intestinal transit time (3.32 h), target Dn (1), the dose, and m_dissolved_ (using the dose for Class I and III APIs; solubility multiplied by 500 mL for Class II and IV). As the usual single doses are known for all three APIs, these doses were chosen for the r_target_ calculations, noting that for compounds in preclinical development, the evaluation would typically be made at the rDCS standard doses of 5, 50 and 500 mg.

## 4. Results

### 4.1. Physicochemical Characterization 

#### 4.1.1. Dissociation Constant (pK_a_) 

Experimental and literature pK_a_ values for voriconazole, lemborexant and istradefylline are summarized in Table 3.

The pK_a_ of lemborexant determined with the Fast UV method was 2.18 ± 0.05, noting that the value was associated with a low absorbance in the assay. Comparative literature values were estimated to be <3.5 by capillary electrophoresis [33].

The experimental pK_a_ values of voriconazole and istradefylline lay outside the standard titration range of pH 2 to 12. In both cases a pK_a_ below the lower calibration limit of the Fast UV method was detected, but not reported. For voriconazole, a basic pK_a_ of 1.76 associated with the triazole moiety has been reported in the literature [32], while for istradefylline a basic pK_a_ of 0.78 has been reported [31,34].

For comparison, in silico predicted pK_a_ values are tabulated in Supplementary Materials, noting that certain values deviated substantially from the values shown in Table 3.

#### 4.1.2. n-Octanol/Water Distribution Coefficient (logD_pH7.4_)

Experimental logD_pH7.4_ and literature logP values for the three APIs are summarized in Table 4. Based on the pK_a_ values discussed above, all compounds are uncharged at pH 7.4. Therefore, the logD_pH7.4_ and logP values are identical. The experimental values differed from those reported in the literature by less than 10% for voriconazole, and less than 25% for lemborexant and istradefylline. In silico predictions of logP can be found in Supplementary Materials. These differed from the experimental values by less than 45%.

### 4.2. rDCS Standard Investigations: Solubility Studies 

The results of the solubility studies in FaSSIF V1, along with the corresponding crystalline forms, are presented in Table 5.

The XRPD patterns and DSC thermograms of the starting materials and the residual solids from the solubility studies were identical for all investigated APIs (Supplementary Materials). The following crystalline forms were identified: form B of voriconazole [35], form CS2 of lemborexant [36], and form I of istradefylline [37,38]. The crystalline forms were consistent with the forms used in the respective marketed formulations [30,34,37,39,40]. 

### 4.3. rDCS Standard Investigations: Caco-2 Permeability Studies

Caco-2 P_app_ values for both transport directions, with and without inhibitor, as well as derived P_eff_ values are summarized in Table 6. Recoveries were >87% (voriconazole), >74% (lemborexant), and >85% (istradefylline). Using antipyrine (P_app_(A⟹B) = 43.9 × 10^−6^ cm/s), propranolol (P_app_(A⟹B) = 13.0 ± 0.7 × 10^−6^ cm/s) and atenolol (P_app_(A⟹B) = 0.197 ± 0.051 × 10^−6^ cm/s) as comparators, all three substances were considered highly permeable. The efflux ratios were less than 2 for all substances, indicating no significant in vitro P-gp transporter effects [41]. P_app_(A⟹B) values were converted to P_eff_ using propranolol and atenolol as calibrators and resulted in similar predicted values for all three substances.

### 4.4. rDCS Customized Investigations: Intrinsic Dissolution Rate 

**Figure 4:**
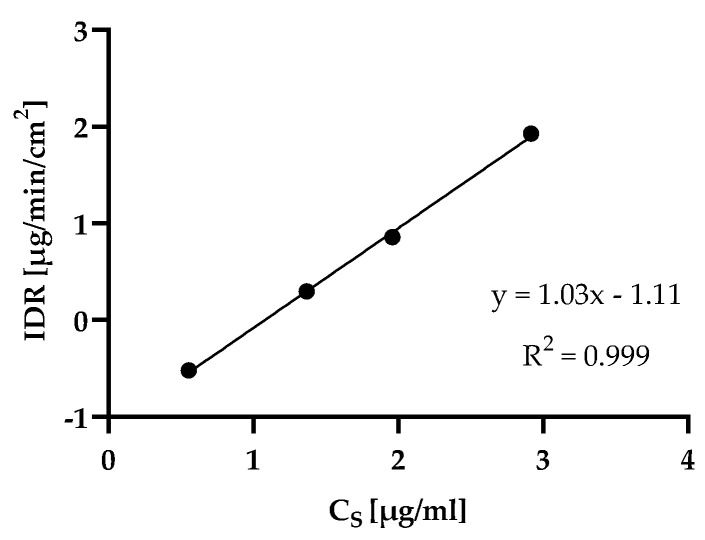
Relationship between IDR in µg/min/cm^2^ and C_S_ in µg/mL (both measured in FaSSIF V1) using logarithmic coordinates. IDRs of voriconazole and lemborexant were measured with the disk dissolution method, the IDR of istradefylline was derived from powder dissolution experiments. Disk IDR and solubility data of an additional API (voxelotor) in the same medium were added to support the correlation. Error bars fall within the data icons.

The results of the IDR studies in FaSSIF V1, including the corresponding crystalline forms, are presented in Table 5. IDR values and thermodynamic solubilities of the three APIs correlated well (Figure 4).

Overlays of the XRPD patterns and the DSC thermograms comparing the starting materials for disk compression and the disks pre- and post-dissolution can be found in Supplementary Materials. The solid state of voriconazole and lemborexant did not change after disk compression and dissolution. Analyses of istradefylline disks using XRPD and DSC indicated a solid state change upon compression. Consequently, the IDR was determined with the powder dissolution method.

### 4.5. rDCS Calculations 

#### 4.5.1. rDCS Standard Investigations

The rDCS classifications of voriconazole, lemborexant and istradefylline according to the standard investigations including SLADs can be found in Table 5.

#### 4.5.2. rDCS Customized Investigations: Dissolution Rate Analysis

The dissolution rate analyses, conducted according to Rosenberger et al., are shown in Table 7 [10,11]. For all APIs, k_diss_ was smaller than k_perm_, with a rank order of k_diss_ (voriconazole) > k_diss_ (lemborexant) > k_diss_ (istradefylline). The predicted risk for dissolution rate limitations therefore increased from voriconazole to lemborexant and istradefylline, in line with the measured thermodynamic solubilities. The calculated r_target_ estimates are displayed in Table 8.

## 5. Discussion

In early preclinical development, pharmaceutical profiling is an important step in the selection of drug candidates and the subsequent formulation campaign. Typically, in vitro characterization of API candidates, early Physiologically based Biopharmaceutics Modeling (PBBM) and comparative PK studies of formulations in animals support biopharmaceutical research at this stage. The main challenge for the pharmaceutical industry at present is to reduce (or even eliminate) animal testing in biopharmaceutical research while ensuring that the formulation for first-in-human studies is appropriate. The rDCS provides rational and science-based guidance to formulators, while maintaining simplicity as a key element to accommodate the demand for medium to high throughput.

One of the key elements of the rDCS is identifying an appropriate particle size for oral formulations. This is of special importance, since particle size not only influences the dissolution behavior and bioavailability of many drug substances, but also impacts processability and critical quality attributes of the final product, such as stability, content uniformity and product appearance [8]. Building on the existing rDCS framework, new equations for the calculation of target particle size are now introduced to more completely explore the risk of incomplete absorption due to dissolution rate issues and facilitate biopharmaceutical decision-making.

### 5.1. Complementarity of the Dissolution/Transit Balance and Dissolution/Permeation Balance Approach

Dissolution/transit balance: Particle size recommendations based on the Dn address how to achieve complete dissolution of the API dose in the intestinal fluid within the intestinal transit time [9]. When Dn = 1, the dose will dissolve within an intestinal transit time of 3.32 h. This approach relies on the assumption of sink conditions and is therefore best suited for APIs that have high permeability and are thus rapidly absorbed once they dissolve and/or have a low D/S ratio. The assumption is less suitable for APIs with solubility-limited absorption (high D/S ratio) and/or a medium to low permeability. To determine how variability in transit time might affect the absorbed fraction, transit times over the usual range of 2–5 h can be explored [42]. If a formulation is required to dissolve within a shorter time to achieve rapid onset, either the transit time can be shortened or the target Dn raised to account for this. In both cases, lower r_target_ values will result. This approach would also be useful for APIs with an absorption window in the intestinal tract [9].

Dissolution/permeation balance: Rosenberger et al. extended the DCS dissolution rate analysis by comparing dissolution (k_diss_) and permeation (k_perm_) rate constants to estimate if the dissolution or permeation rate represents the limiting step in oral drug absorption [10,11]. However, equality of the rate constants does not necessarily mean that the dissolution and permeation rates are the same: whereas the main driving force for dissolution is the solubility at the surface of the API, the driving force for permeation is the concentration of the API already dissolved in the intestinal lumen. Although the upper limit of the luminal concentration is the solubility of the API, luminal concentrations may be much lower if the permeability is high or if the applied dose does not saturate the luminal fluids. The rDCS authors addressed these considerations by highlighting that useful risk classifications can only be derived when k_diss_ >> k_perm_ or k_diss_ << k_perm_ [10]. Another limitation of the rate constant comparison is that k_diss_ must be calculated with a specific particle size, which was set to r = 100 µm [10,11].

In the present work, the estimated maximal rates of dissolution and permeation rather than their rate constants are compared. This approach offers the advantage of taking the main driving forces for dissolution and permeation into account while delivering a specific target particle size. It thereby complements the dissolution/transit balance approach by relating r_target_ values to permeability. The r_target_ values can be interpreted as the particle size threshold above which dissolution rather than the permeation becomes limiting to oral absorption.

### 5.2. Use of Solubility and Intrinsic Dissolution Rate as Input for Target Particle Size Calculations 

The DCS recommended an estimate of the intestinal solubility to calculate r_target_ based on Dn. In the rDCS the IDR was added as a further possibility for calculating the dissolution rate constant. This work explored both input parameters. Comparison of the results obtained using C_S_ and IDR can help to inform the formulator whether factors other than the solubility influence the dissolution rate, e.g., wettability of the surface or simultaneous reactions during diffusion across the ABL [10,43,44,45,46,47,48,49]. Possible reactions include acid-base reactions (ionization), partitioning into bile component micelles (or those generated by adding surfactant excipients), or complexation [43,44,45,46,47,48,49,50]. For instance, Teleki et al. reported that IDR values were not solely determined by solubility and its pH dependence because several APIs with similar solubilities in FaSSIF V1 and fed state simulated intestinal fluid (FeSSIF) showed notable differences in their IDRs in the same media [46]. 

We note that, for poorly soluble weak bases, the solubility and IDR determined under intestinal conditions represent a “worst case” (most conservative) scenario, because they assume that there is no supersaturation in the intestine [9]. For weak bases, calculating the r_target_ values using the gastric solubility (or IDR) as input (“best case” scenario) or supersaturation data from two-stage or transfer model experiments, as suggested in the rDCS customized investigations, may help to clarify the potential in vivo performance of the API, and set formulation goals.

### 5.3. Selection of the Relevant Aqueous Boundary Layer Assumption 

Assumptions about the ABL thickness (h_particle_) are required in all previously described approaches. In the DCS and rDCS approaches, h_particle_ was set equal to the particle radius [9]. As described earlier, this assumption is generally thought to be most relevant for particle radii < 30 µm [19]. For particle sizes exceeding 30 µm, h_particle_ tends to approach a constant value and can be set to, e.g., 30 µm or, alternatively, h_disk_. In the present work, the choice of h_particle_ was therefore addressed by implementing a bracketing approach using multiple equations that represent “best case” and “worst case” scenarios. The r_target_ values can be calculated under the following h assumptions: (i) h_particle_ = r_0_, (ii) h_particle_ = 30 µm, and (iii) h_particle_ = h_disk_. It is recommended to calculate the r_target_ values using all three assumptions, and if the target value calculated using h_particle_ = r_0_ is greater than 30 µm, put more weight on the values calculated using the 30 µm or h_disk_ assumptions. 

### 5.4. Case Studies

Voriconazole, lemborexant and istradefylline were used as model drugs for implementation of the various approaches to calculating r_target_, since they represent compounds with widely different chemical structures, solubilities, and IDRs, which are reflected in their respective rDCS classifications and r_target_ estimations (Table 5 and Table 8). 

#### 5.4.1. Voriconazole

Voriconazole is a weakly basic and moderately lipophilic API with a pK_a_ of 1.76 [32] and a logD_pH7.4_ of 1.66 ± 0.03. It is predominantly unionized under physiological conditions in the small intestine (pH 6.5) and has a measured solubility in FaSSIF V1 of 827 ± 6 µg/mL. The measured disk IDR (84.8 ± 1.4 µg/min/cm^2^) correlated well with the theoretical disk IDR (based on the Levich equation with C_S_ as input) of 87.0 ± 0.6 µg/min/cm^2^ [23]. This suggests that the disk dissolution rate was not impacted by factors such as wetting or reactions in the ABL. As a result, r_target_ estimates calculated from C_S_ and IDR were not significantly different (*p* = 0.659). The human P_eff_ of 4.30 × 10^−6^ cm/s, estimated from in vitro Caco-2 permeability data, suggests high permeability of the compound (Table 6). 

Doses up to 414 mg (D/S ratio = 500 mL) were classified as rDCS class I, while higher doses up to the SLAD of 1810 mg were classified as class IIa. In healthy volunteers, typical therapeutic doses of voriconazole, such as 200 mg (oral maintenance dose) or 400 mg (oral loading dose), are well absorbed with bioavailabilities of 85% or higher [29,51]. This is in line with the low risk predicted by the rDCS standard investigations (rDCS class I_200mg_ and I_400mg_). After administration of high single oral doses of 800, 1200 and 1600 mg, voriconazole exposure in terms of AUC and C_max_ increased proportionally with dose, in line with the calculated SLAD [52]. The low developability risk for voriconazole predicted by the rDCS standard investigations was thus in line with the clinical findings. 

Regarding target particle size, the dissolution/transit balance approach with h_particle_ = r_0_ and C_S_ as input (Equation (7)) resulted in a r_target_ value of 124 µm. As mentioned above, when target particle size values based on h_particle_ = r_0_ exceed 30 µm, this approach might be too conservative. The other h_particle_ assumptions (h_particle_ = 30 µm or h_particle_ = h_disk_ = 42.5 µm) resulted in larger r_target_ estimates of 516 µm and 364 µm, respectively. In summary, the dissolution/transit balance approach indicated that there is no need for extensive particle size reduction when formulating a conventional oral dosage form containing voriconazole. The dissolution/permeation balance approach with C_S_ as input led to a similar conclusion (r_target_ = 118 µm with h_particle_ = 30 µm and r_target_ = 83.3 µm with h_particle_ = h_disk_ = 42.5 µm), although the particle size limit was lower across all ABL assumptions. Voriconazole is an rDCS class I drug, i.e., it is highly permeable and has a low D/S ratio at the recommended dose levels, so that sink conditions will likely be maintained during in vivo dissolution. Therefore, the dissolution/transit balance approach is expected to yield the most relevant values.

Relevant information on voriconazole particle size in products is scarce in the open literature. The EMA European Public Assessment report (EPAR) for the originator VFEND^®^ stated that batches with a wide variety of particle size distributions were clinically acceptable [53]. Generic film-coated tablets manufactured from drug substance with D_90_ ≤ 90 μm (Vorcon, Southern Cross Pharma Pty Ltd., Hawthorn, Australia) were bioequivalent to the innovator VFEND^®^ (200 mg) and both Vorcon and VFEND^®^ tablets dissolved to an extent of ≥85% within 15 min in “media of various pH” [54]. The in vitro dissolution profiles of tablets containing unmicronised API (particle size not disclosed) were reported to be noticeably slower [54]. As the particle size specification for the Vorcon product is below the estimated r_target_ values discussed above, it falls well within a “safe space” in which dissolution rate limitations to oral absorption are very unlikely to occur.

#### 5.4.2. Lemborexant

Lemborexant is a lipophilic weak base with a pK_a_ of 2.18 ± 0.05 and a logD_pH7.4_ of 3.04 ± 0.05. Under small-intestinal pH conditions lemborexant is present in its neutral form and exhibits poor solubility (23.3 ± 0.1 µg/mL). The measured disk IDR in FaSSIF V1 (1.99 ± 0.09 µg/min/cm^2^) was slightly lower than the theoretical IDR (2.24 ± 0.01 µg/min/cm^2^) estimated from C_S_ (*p* = 0.014). Although statistically significant, the difference in r_target_ values calculated from C_S_ or IDR did not seem to be of practical relevance (Table 8). Lemborexant was considered highly permeable with a predicted human P_eff_ of 4.20 × 10^−6^ cm/s. The rDCS classified lemborexant as follows: rDCS class I_5mg_, IIa/b_50mg_ and IIb_500mg_ with an SLAD of 49.6 mg.

Tablets for oral use containing 2.5, 5, or 10 mg of lemborexant are commercially available [55]. In line with its classification as rDCS class I_1-10mg_, single oral lemborexant doses ranging from 1 to 10 mg were absorbed rapidly in healthy adults with a median t_max_ of approximately 1 h [56,57]. In a human mass-balance study only about 13% of a 10 mg [^14^C]lemborexant dose was recovered unchanged in feces, corresponding to a fraction of oral dose absorbed (Fa) of 87% [33,57,58]. Lemborexant exposure was shown to increase in a dose-proportional manner in the range 1 to 10 mg [56].

The dissolution rate analysis for the 10 mg dose resulted in particle size estimates <30 µm (Table 8), so h_particle_ = r_0_ likely represents the most relevant assumption of ABL thickness. Values using h_particle_ = 30 µm or h_particle_ = h_disk_ = 40.7 µm led to lower r_target_ values since in these approaches, h_particle_ was larger than the calculated particle radius. In summary, the dissolution/transit balance approach predicted particles with a radius of ≤20 µm to dissolve within the intestinal transit time under sink conditions and the dissolution/permeation balance approach predicted a particle size cut-off for dissolution rate limitations of approximately 10 µm. Like voriconazole, lemborexant is an rDCS Class I drug at the commercially available dose levels and hence in vivo dissolution under sink conditions can be expected. Therefore, r_target_ values estimated using the dissolution/transit balance approach are likely to be more relevant than values derived from the dissolution/permeation balance approach. 

**Figure 5:**
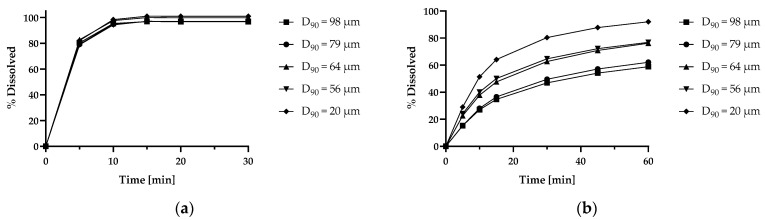
Particle size dependent dissolution profiles of 10 mg lemborexant tablets in (**a**) 0.1 M hydrochloric acid, and (**b**) pH 6.8 (Japanese Pharmacopoeia). Adapted from ref. [59].

Particle size specifications of the marketed product (DAYVIGO^®^) have not been disclosed. However, dissolution of lemborexant tablets containing 2.5 to 10 mg API has been reported in the patent literature [59]. The dissolution curves for 10 mg lemborexant tablets with different D_90_ values are presented in Figure 5. Lemborexant dissolution was reported to be fast and not affected by particle size in the range of 20 to 98 µm (D_90_) in 0.1 M hydrochloric acid [59], where the solubility of lemborexant is considerably enhanced by ionization (1.6 mg/mL at pH 1.0) [39]. Dissolution was slower and substantially affected by particle size when tested at pH 6.8, in line with the target particle sizes of ≤20 µm calculated according to the equations using the dissolution/transit balance approach (Table 8) [59]. 

#### 5.4.3. Istradefylline 

The xanthine derivative istradefylline is a moderately lipophilic (logD_pH7.4_ = 2.96 ± 0.03) and very weakly basic API (pK_a_ = 0.78) [31,34] exhibiting poor solubility in FaSSIF V1 (3.59 ± 0.05 µg/mL). The IDR of istradefylline estimated from powder dissolution experiments in FaSSIF V1 (0.302 ± 0.010 µg/min/cm^2^) was somewhat lower than the value estimated from its solubility (0.346 ± 0.005 µg/min/cm^2^, *p* = 0.0009). As for lemborexant, the difference had no practical impact on the r_target_ estimations (Table 8). The very low solubility combined with the high estimated human P_eff_ of 4.66 × 10^−6^ cm/s resulted in classification of istradefylline as rDCS class IIa_5mg_, IIb_50mg_ and IIb_500mg_ with an SLAD of 8.50 mg.

The rDCS and r_target_ analyses indicated that the development of conventional oral istradefylline formulations at doses of around 10 mg or more would be associated with a high risk, much greater than for voriconazole or lemborexant. The classification as class IIb indicated that the solubility of the API poses a potential risk, and the r_target_ estimations showed that dissolution rate can have an additional influence. It is noted that particle size estimates using the dissolution/transit balance approach tend to lose some relevance for rDCS class IIb drugs because sink conditions are likely not maintained during the intestinal transit (due to their high D/S ratios). However, the r_target_ values calculated from the dissolution/permeation balance approach suggest that particle size reduction below approximately 12 µm may help mitigate dissolution rate limitations.

Oral tablets containing istradefylline are marketed in Japan (20 mg) and the USA (20 and 40 mg) [60,61]. The 20 and 40 mg tablets are dose proportional [34]. To the best of our knowledge, clinical studies investigating the absolute oral bioavailability of istradefylline formulations have not been conducted because of the compound’s poor aqueous solubility, which has prevented the development of a suitable intravenous formulation for clinical use [62]. However, after oral administration of a suspension containing 40 mg of [^14^C]istradefylline to healthy male subjects, 38.9 ± 10.7% and 48.0 ± 13.4% of the administered radioactivity were recovered in urine and feces, respectively [63]. The significant amount of drug in feces suggests that istradefylline absorption may be incomplete, in line with its classification as an rDCS class IIb API.

**Figure 6:**
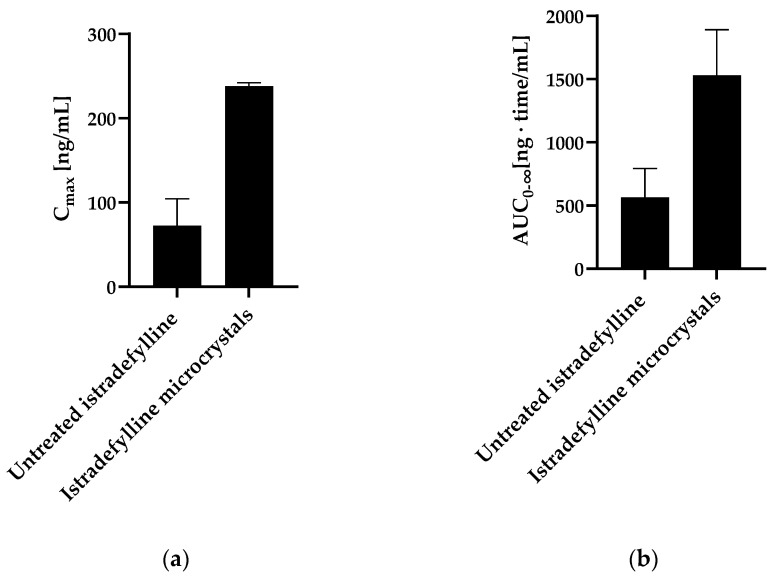
(**a**) C_max_, and (**b**) AUC of unpulverized istradefylline (average particle size = 181 µm) and istradefylline microcrystals (average particle size = 11 µm) suspended in 0.5 *w*/*v* % methylcellulose aqueous vehicle (0.3 mg/mL) and orally administered to male rats (body weight: 209 to 233 g) at the dose of 10 mL/kg. Data are presented as the mean ± SEM. Adapted from ref. [64].

Particle size reduction of istradefylline to particle radii ≤12.6 µm (h_particle_ = r_0_) was expected to reduce the risk of dissolution rate limitations at the 20 mg dose level. However, particle size reduction alone is not expected to resolve the solubility issue, which should be addressed through other formulation approaches. According to the patent literature, crystals of istradefylline exhibit low aqueous solubility and are needle-like with a width of several µm and a length of several hundred µm (Supplementary Materials) [64]. Particle size reduction of istradefylline crystals was performed, resulting in microcrystals with an average particle size of 0.5 to 20 µm and an increased amorphous content of up to 80% [64]. The reduction in particle size and generation of amorphous material resulted in a significant increase in the dissolution rate of the microcrystals in water [64]. Although there are no supporting human data available, administration of a suspension of the microcrystals resulted in an approximately three-fold increase in both AUC and C_max_ in rats compared to the unmilled material (Figure 6) [64]. This example supports the use of the rDCS and r_target_ calculations to estimate the risks associated with istradefylline formulations and guide the efforts necessary to guarantee sufficient bioavailability by combining the strategies of particle size reduction to increase dissolution rate and amorphization to increase solubility.

## 6. Conclusions

This work introduces new, analytically solvable equations for estimating API target particle size based on in vitro tests that are commonly used in early oral formulation development (solubility, permeability, IDR). An Excel spreadsheet for automated calculation of all r_target_ values is provided as Supplementary Materials for easy implementation of the equations.

As demonstrated with the three case examples presented, this rDCS-based approach can be used to support initial particle size specifications, suggest whether particle size reduction may be an appropriate formulation strategy and determine whether a formulation which enhances solubility is required. 

Further benefits of this approach are expected at various stages of oral drug development, including (i) selection of a suitable particle size for preclinical toxicological studies, thus reducing the use of animals in this type of study as well as in formulation assessment, (ii) a priori selection of a suitable particle size for first-in-human studies and thus reduction or even elimination of the need for pharmacokinetic studies in large animal species such as dogs and swine, and (iii) rational assessment of the absorption risk linked to API particle size, which can then trigger the investigation of clinically relevant specifications at the time of filing.

Application of the r_target_ equations to more drugs with diverse structures and properties will assist in establishing them as a practical substitute for trial-and-error approaches for determining drug particle size in preclinical toxicity studies as well as reducing the use of animals and time required for formulation development.

## Figures and Tables

**Table 1:** Key assumptions and their mathematical formulation for the derivation of the dissolution rate constant (k_diss_) from the Noyes–Whitney/Nernst–Brunner equation.

Assumption		Mathematical Formulation
1	Total powder surface area of a monodispersed spherical particle population ^1^ [16]	$A_{powder}=A_{p}\cdot N_{0}=4\pi {r_{0}}^{2}\cdot \frac{Dose}{\frac{4}{3}\pi {r_{0}}^{3}\rho }=\frac{3\cdot Dose}{r_{0}\cdot \rho }$
2	Dissolution under sink conditions	$C_{S}-C_{t}=C_{S}$ i.e., $C_{t}=0$
3	Aqueous boundary layer (ABL) thickness equals initial particle radius for particles with r_0_ < 30 µm [17]	$h_{particle}=r_{0}$
	ABL thickness equals constant value for particles with r_0_ ≥ 30 µm [3,18,19,20]	e.g., $h_{particle}=30\;µm$

^1^ where A_p_ is the surface area of a single particle and N_0_ is the number of particles.

**Table 2:** Summary of r_target_ equations applying different approaches and h_particle_ assumptions.

Approach		Based on	r_target_ Equation			
			h_particle_ = r_0_		h_particle_ ≠ r_0_	
2.1	Dissolution/ Transit balance	Dn and C_S_	Equation (7) ^1^:	$\sqrt{\frac{3\cdot D\cdot C_{s}\cdot T_{si}}{{Dn}_{target}\cdot \rho }}$	Equation (8) ^2^:	$\frac{3\cdot D\cdot C_{s}\cdot T_{si}}{{Dn}_{target}\cdot \rho \cdot h_{particle}}$
2.2		Dn and IDR	Equation (14) ^2^:	$\sqrt{\frac{3\cdot h_{disk}\cdot IDR\cdot T_{si}}{{Dn}_{target}\cdot \rho }}$	Equation (15) ^2^:	$\frac{3\cdot h_{disk}\cdot IDR\cdot T_{si}}{{Dn}_{target}\cdot \rho \cdot h_{particle}}$
2.3	Dissolution/ Permeation balance	k_diss_/k_perm_ and C_S_	Equation (20) ^2^:	$\sqrt{\frac{3\cdot D\cdot Dose{\cdot R}_{si}{\cdot C}_{s}}{DF\cdot P_{eff}\cdot \rho {\cdot m}_{dissolved}}}$	Equation (21) ^2^:	$\frac{3\cdot D\cdot Dose\cdot R_{si}{\cdot C}_{s}}{DF\cdot P_{eff}\cdot \rho {\cdot m}_{dissolved}\cdot h_{particle}}$
2.4		k_diss_/k_perm_ and IDR	Equation (22) ^2^:	$\sqrt{\frac{3\cdot h_{disk}\cdot Dose\cdot R_{si}\cdot IDR}{DF\cdot P_{eff}\cdot \rho {\cdot m}_{dissolved}}}$	Equation (23) ^2^:	$\frac{3\cdot h_{disk}\cdot Dose\cdot R_{si}\cdot IDR}{DF\cdot P_{eff}\cdot \rho {\cdot m}_{dissolved}\cdot h_{particle}}$
2.5		A_powder_ and IDR	-		Equation (26) ^2^:	$\frac{3\cdot Dose\cdot R_{si}\cdot IDR}{DF\cdot P_{eff}\cdot \rho \cdot m_{dissolved}}$

^1^ Ref. [9].^2^ Novel r_target_ equation.

**Table 3:** Experimental and literature pK_a_ values of voriconazole, lemborexant and istradefylline.

Method for pK_a_ Determination	Voriconazole	Lemborexant	Istradefylline
Fast UV (Section 3.2.1)	- ^1^	2.18 ± 0.05 ^2^	- ^1^
Literature pK_a_	1.76 ^3^ [32]	<3.50 [33]	0.78 ^3^ [31,34]

^1^ Evidence of additional low pK_a_ outside the calibration limit (pH 2 to 12). ^2^ Low absorbance (<0.30 abs units) associated with the pK_a_, value should be treated with caution. ^3^ Method of determination not disclosed.

**Table 4:** Experimental and literature logD_pH7.4_/logP ^1^ of voriconazole, lemborexant and istradefylline.

Method for logD_pH7.4_/logP Determination	Voriconazole	Lemborexant	Istradefylline
Shake-flask logD_pH7.4_	1.66 ± 0.03	3.04 ± 0.05	2.96 ± 0.03
Literature logP ^2^	1.80 [32]	3.70 [33]	3.5–3.6 [34]

^1^ As none of the three compounds are ionized at pH 7.4, the logP and logD_pH7.4_ are identical. ^2^ Method of determination not disclosed.

**Table 5:** Results of the thermodynamic solubility and IDR studies including solid state analyses, SLADs and rDCS classifications for 5, 50, 500 mg and a usual dose.

API	C_S_ ^1^ FaSSIF V1 [µg/mL]	IDR ^a^ FaSSIF V1 [µg/min/cm^2^]	Cryst. Form	SLAD	rDCS Classification			
					5 mg	50 mg	500 mg	Usual dose
Voriconazole	827 ± 6	84.8 ± 1.4 ^2^	B ^4^	1810	I	I	IIa	I_200mg_
Lemborexant	23.3 ± 0.1	1.99 ± 0.09 ^2^	CS2 ^4^	49.6	I	IIa/b	IIb	I_10mg_
Istradefylline	3.59 ± 0.05	0.302 ± 0.010 ^3^	I ^5^	8.50	IIa	IIb	IIb	IIb_20mg_

^1^ pH 6.50 ± 0.05. ^2^ Measured with the disk dissolution method. ^3^ Measured with the powder dissolution method. ^4^ Based on the solid state analysis after the solubility study, disk compression, and disk dissolution. ^5^ Based on the solid state analysis after the solubility study. ^a^ Intrinsic dissolution rate

**Table 6:** Measured Caco-2 P_app_ and derived P_eff_ values for voriconazole, lemborexant and istradefylline.

Permeability Measure		Voriconazole	Lemborexant	Istradefylline
P_app_(A⟹B)	[×10^−6^ cm/s]	27.3 ± 1.7	26.7 ± 1.2	30.2 ± 1.2
P_app_(A⟹B) + inhibitor	[×10^−6^ cm/s]	24.0 ± 1.0	24.2 ± 0.7	31.2 ± 1.1
P_app_(B⟹A)	[×10^−6^ cm/s]	43.5 ± 0.9	41.8 ± 1.2	28.3 ± 16.0
P_app_(B⟹A) + inhibitor	[×10^−6^ cm/s]	40.4 ± 1.1	39.0 ± 0.4	43.7 ± 18.5
Estimated human P_eff_	[×10^−4^ cm/s]	4.30	4.20	4.66

**Table 7:** Results for k_diss_ and k_perm_ using the rDCS approach by Rosenberger et al. [10,11].

		Voriconazole	Lemborexant	Istradefylline
k_diss_	[min^−1^]	7.56 × 10^−3^	1.80 × 10^−4^	2.73 × 10^−5^
k_perm_	[min^−1^]	2.19 × 10^−2^	2.14 × 10^−2^	2.38 × 10^−2^

**Table 8:** Calculated r_target_ values for voriconazole, lemborexant and istradefylline using the approaches shown in Figure 1.

Approach	Based on	r_target_ [µm]								
		Voriconazole (200 mg)			Lemborexant (10 mg)			Istradefylline (20 mg)		
		h_particle_ = r_0_	h_particle_ = 30 µm	h_particle_ = h_disk_ ^1^	h_particle_ = r_0_	h_particle_ = 30 µm	h_particle_ = h_disk_ ^1^	h_particle_ = r_0_	h_particle_ = 30 µm	h_particle_ = h_disk_ ^1^
Dissolution/ transit balance	Dn and C_S_	124	516	364	20.1	13.5	9.94	8.23	2.26	1.67
	Dn and IDR	123	502	354	18.9	11.9	8.81	7.69	1.97	1.46
Dissolution/ permeation balance	k_diss_/k_perm_ and C_S_	59.5	118	83.3	9.73	3.16	2.33	12.6	5.32	3.92
	k_diss_/k_perm_ and IDR	58.7	115	81.0	9.16	2.80	2.06	11.8	4.64	3.43
	A_powder_ and IDR	-		81.0	*-*		2.06	-		3.43

^1^ h_disk_ = 42.5 µm (voriconazole), 40.7 µm (lemborexant), 40.7 µm (istradefylline).

## Data Availability

All data is reported in the manuscript and in the Supplementary Materials.

## Supplementary Materials

The following supporting information can be downloaded at: https://www.mdpi.com/article/10.3390/pharmaceutics15071909/s1, File S1: Projection of target drug particle size; File S2: Template for Automated r_target_ Calculations. References [3,4,5,6,7,10,12,16,17,18,19,20,23,25,26,31,32,33,34,35,41,65,66] are cited in the Supplementary Materials.
