# Supplementary material for: Projection of Target Drug Particle Size in Oral Formulations Using the Refined Developability Classification System (rDCS)
Source: Pharmaceutics. 2023 Jul 8;15(7):1909. doi: 10.3390/pharmaceutics15071909 (PMC10385664; doi:10.3390/pharmaceutics15071909)
Supplement: Supplementary file 1 [file pharmaceutics-15-01909-s001.zip › File S1. Projection of target drug particle size_proofread.pdf]

Projection of Target Drug Particle Size in Oral Formulations Using the Refined Developability Classification System (rDCS)

Kristian Beran [1,2], Eline Hermans [2], René Holm [3], Kia Sepassi [4] and Jennifer Dressman [1,*]

[1] Fraunhofer Institute of Translational Medicine and Pharmacology, 60596 Frankfurt am Main, Germany
[2] Janssen Research & Development, Pharmaceutical & Material Sciences, 2340 Beerse, Belgium
[3] Department of Physics, Chemistry and Pharmacy, University of Southern Denmark, 5230 Odense, Denmark
[4] Janssen Research & Development, Discovery Pharmaceutics, La Jolla, CA 92121, USA
* Correspondence: Jennifer.Dressman@itmp.fraunhofer.de; Tel.: +49-(0)-69-7438-6408

Supplementary Materials

S.1. Theoretical Section

S.1.1. Dissolution/Transit Balance: Derivation of k_{diss} based on Solubility

The Noyes-Whitney/Nernst-Brunner equation can be expressed as follows (Equation ((S1))):

$$\frac{dM}{dt} = \frac{D}{h_{particle}} \cdot A_{powder} \cdot (C_S - C_t) \qquad \text{(S1)}$$

where dM/dt is the dissolution rate, D is the diffusion coefficient, $h_{particle}$ is the thickness of the aqueous boundary layer (ABL) next to the particle of interest, A_{powder} is the powder surface area, C_S is the thermodynamic solubility and C_t is the API concentration at time t [1-4]. The three key assumptions summarized in Table S1 (corresponding to Table 1 in the main text) can be incorporated into Equation (S1) to derive the dissolution rate constant (k_{diss}).

Table S1. Key assumptions and their mathematical formulation for the derivation of the dissolution rate constant (k_{diss}) from the Noyes-Whitney/Nernst-Brunner equation.

	Assumption	Mathematical formulation
1	Total powder surface area of a monodispersed spherical particle population [1] [5]	$A_{powder} = A_p \cdot N_0 = 4\pi {r_0}^2 \cdot \frac{Dose}{\frac{4}{3}\pi {r_0}^3 \rho} = \frac{3 \cdot Dose}{r_0 \cdot \rho}$
2	Dissolution under sink conditions	$C_S - C_t = C_S$ i.e., $C_t = 0$
3	Aqueous boundary layer (ABL) thickness equals initial particle radius for particles with $r_0 < 30$ µm [6]	$h_{particle} = r_0$
	ABL thickness equals constant value for particles with $r_0 \geq 30$ µm [7-10]	e.g., $h_{particle} = 30$ µm

[1] where A_p is the surface area of a single particle and N_0 is the number of particles

Inclusion of assumption 1 about A_{powder} into Equation (S1) leads to Equation (S2):

$$\frac{dM}{dt} = \frac{D}{h_{particle}} \cdot \frac{3 \cdot Dose}{r_0 \cdot \rho} \cdot (C_S - C_t) \qquad \text{(S2)}$$

Equation (S3) can be obtained after incorporation of the sink conditions assumption (assumption 2):

$$\frac{dM}{dt} = \frac{D}{h_{particle}} \cdot \frac{3 \cdot Dose}{r_0 \cdot \rho} \cdot C_S \qquad \text{(S3)}$$

Rearrangement of Equation (S3) leads to a dissolution rate equation (Equation (S4)), which defines k_{diss} (with variable h). This k_{diss} definition is presented in the main text as Equation (6).

$$\frac{dM}{dt} = \frac{3 \cdot D}{r_0 \cdot h_{particle}} \cdot \frac{C_S}{\rho} \cdot Dose = k_{diss} \cdot Dose \qquad (r_0 \geq 30\ \mu m,\ h_{particle} = 30\ \mu m) \qquad (S4)$$

Incorporating assumption 3 for particle radii smaller than 30 μm ($h_{particle} = r_0$) results in Equation (S5) with a different k_{diss} term, corresponding to Equation (5) in the main text.

$$\frac{dM}{dt} = \frac{3 \cdot D}{{r_0}^2} \cdot \frac{C_S}{\rho} \cdot Dose = k_{diss} \cdot Dose \qquad (r_0 < 30\ \mu m,\ h_{particle} = r_0) \qquad (S5)$$

Therefore, k_{diss} based on C_S can be calculated as follows (Equations (S6) and (S7)):

$$k_{diss} = \frac{3 \cdot D}{{r_0}^2} \cdot \frac{C_S}{\rho} \qquad (r_0 < 30\ \mu m,\ h_{particle} = r_0) \qquad (S6)$$

$$k_{diss} = \frac{3 \cdot D}{r_0 \cdot h_{particle}} \cdot \frac{C_S}{\rho} \qquad (r_0 \geq 30\ \mu m,\ h_{particle} = 30\ \mu m) \qquad (S7)$$

S.1.2. Dissolution/Transit Balance: Derivation of Target Particle Size Equations based on the Dissolution Number and Solubility

The k_{diss} equations derived in Section S.1.1. can be utilized to calculate the Dn according to Equations (S8) and (S9):

$$Dn = \frac{3 \cdot D}{{r_0}^2} \cdot \frac{C_S}{\rho} \cdot T_{si} \qquad (r_0 < 30\ \mu m,\ h_{particle} = r_0) \qquad (S8)$$

$$Dn = \frac{3 \cdot D}{r_0 \cdot h_{particle}} \cdot \frac{C_S}{\rho} \cdot T_{si} \qquad (r_0 \geq 30\ \mu m,\ h_{particle} = 30\ \mu m) \qquad (S9)$$

The rearrangement for r_0 and application of a target Dn leads to the r_{target} equations (Equations (S10) and (S11)) presented in the main text as Equations (7) and (8).

$$R_{target} = \sqrt{\frac{3 \cdot D \cdot C_S \cdot T_{si}}{Dn_{target} \cdot \rho}} \qquad (r_0 < 30\ \mu m,\ h_{particle} = r_0) \qquad (S10)$$

$$r_{target} = \frac{3 \cdot D \cdot C_S \cdot T_{si}}{Dn_{target} \cdot \rho \cdot h_{particle}} \qquad (r_0 \geq 30\ \mu m,\ h_{particle} = 30\ \mu m) \qquad (S11)$$

S.1.3. Dissolution/Transit Balance: Derivation of k_{diss} based on Intrinsic Dissolution Rate

In addition to Equation (S1) (utilizing h_{disk} and A_{disk} rather than $h_{particle}$ and A_{powder}), the Levich equation (Equation (S12)) [11] applies for the dissolution from a rotating disk with a constant surface area under sink conditions and the assumption of convective diffusion without reaction as dissolution mechanism:

$$IDR = 0.62 \cdot D^{\frac{2}{3}} \cdot \nu^{-\frac{1}{6}} \cdot \omega^{\frac{1}{2}} \cdot C_S \qquad (S12)$$

where IDR is the intrinsic dissolution rate, D is the diffusion coefficient, ν is the kinematic viscosity, ω is the rotational velocity and C_S is the thermodynamic solubility. The equation can be rewritten to estimate the IDR using the rotational speed of the compressed disk in revolutions per minute (Equation (S13**Error! Reference source not found.**)). For this, a unit conversion factor of 30/π must be applied for the conversion of ω in rad·s^{-1} to revolutions per minute (RPM) in min^{-1}.

$$\mathrm{IDR} = 0.20 \cdot D^{\frac{2}{3}} \cdot \nu^{-\frac{1}{6}} \cdot \mathrm{RPM}^{\frac{1}{2}} \cdot C_S \tag{S13}$$

The dissolution rate from the disk surface can be calculated via multiplication of the IDR by the disk surface area A_{disk} (Equation (S14)):

$$\frac{dM}{dt} = \mathrm{IDR} \cdot A_{disk} = 0.20 \cdot D^{\frac{2}{3}} \cdot \nu^{-\frac{1}{6}} \cdot \mathrm{RPM}^{\frac{1}{2}} \cdot A_{disk} \cdot C_S \tag{S14}$$

Equation (S1) (adjusted to the dissolution from a disk surface) and Equation (S14) (Levich equation) were equated to obtain Equation (S15).

$$\left(\frac{dM}{dt}\right)_{disk} = \frac{D}{h_{disk}} \cdot A_{disk} \cdot C_S = 0.20 \cdot D^{\frac{2}{3}} \cdot \nu^{-\frac{1}{6}} \cdot \mathrm{RPM}^{\frac{1}{2}} \cdot A_{disk} \cdot C_S \tag{S15}$$

Rearrangement of Equation (S15) for h_{disk} results in an equation for the thickness of the aqueous boundary layer (ABL) of the rotating disk (h_{disk}) under sink conditions (Equation (S16)):

$$h_{disk} = 4.98 \cdot D^{\frac{1}{3}} \cdot \nu^{\frac{1}{6}} \cdot \mathrm{RPM}^{-\frac{1}{2}} \tag{S16}$$

Rosenberger et al. introduced the constant k_{IDR} to calculate k_{diss} for a monodispersed particle population from the disk IDR (Equation (S17)) [12]:

$$k_{diss} = k_{IDR} \cdot \mathrm{IDR} \tag{S17}$$

Equations for k_{IDR} were derived from Equation (S17) (see Equations (S18) and (S19)). The IDR was substituted by the Levich equation (Equation (S13)) and the k_{diss} equations derived in Section S.1.1. were used.

$$k_{diss} = k_{IDR} \cdot 0.20 \cdot D^{\frac{2}{3}} \cdot \nu^{-\frac{1}{6}} \cdot \mathrm{RPM}^{\frac{1}{2}} \cdot A_{disk} \cdot C_S = \frac{3D}{r_0^2} \cdot \frac{C_S}{\rho} \quad (r_0 < 30\ \mu m,\ h_{particle} = r_0) \tag{S18}$$

$$k_{diss} = k_{IDR} \cdot 0.20 \cdot D^{\frac{2}{3}} \cdot \nu^{-\frac{1}{6}} \cdot \mathrm{RPM}^{\frac{1}{2}} \cdot A_{disk} \cdot C_S = \frac{3D}{r_0 \cdot h_{part.}} \cdot \frac{C_S}{\rho} \quad (r_0 \geq 30\ \mu m,\ h_{particle} = 30\ \mu m) \tag{S19}$$

Rearrangement for k_{IDR}, considering Equation (S16) for h_{disk}, resulted in Equations (S20) and (S21):

$$k_{IDR} = \frac{3 \cdot h_{disk}}{r_0{}^2 \cdot \rho} \qquad (r_0 < 30\ \mu m,\ h_{particle} = r_0) \qquad (S20)$$

$$k_{IDR} = \frac{3 \cdot h_{disk}}{r_0 \cdot h_{particle} \cdot \rho} \qquad (r_0 \geq 30\ \mu m,\ h_{particle} = 30\ \mu m) \qquad (S21)$$

Insertion of the k_{IDR} equations (Equations (S20) and (S21)) into Equation (S17) results in the k_{diss} equations based on the IDR (Equations (S22) and (S23)) presented in the main text as Equations (9) and (10).

$$k_{diss} = \frac{3 \cdot h_{disk}}{r_0{}^2 \cdot \rho} \cdot IDR \qquad (r_0 < 30\ \mu m) \qquad (S22)$$

$$k_{diss} = \frac{3 \cdot h_{disk}}{r_0 \cdot h_{particle} \cdot \rho} \cdot IDR \qquad (r_0 \geq 30\ \mu m) \qquad (S23)$$

S.1.4. Dissolution/Transit Balance: Derivation of Target Particle Size Equations based on the Dissolution Number and Intrinsic Dissolution Rate

The k_{diss} equations based on the IDR (Equations (S22) and (S23)) were utilized to calculate the Dn according to Equations (S24) and (S25):

$$Dn = \frac{3 \cdot h_{disk}}{r_0{}^2 \cdot \rho} \cdot IDR \cdot T_{si} \qquad (r_0 < 30\ \mu m,\ h_{particle} = r_0) \qquad (S24)$$

$$Dn = \frac{3 \cdot h_{disk}}{r_0 \cdot h_{particle} \cdot \rho} \cdot IDR \cdot T_{si} \qquad (r_0 \geq 30\ \mu m,\ h_{particle} = 30\ \mu m) \qquad (S25)$$

The rearrangement for r_0 and application of a target Dn leads to the r_{target} equations (Equations (S26) and (S27)) presented in the main text (Equations (14) and (15)):

$$r_{target} = \sqrt{\frac{3 \cdot h_{disk} \cdot IDR \cdot T_{si}}{Dn_{target} \cdot \rho}} \qquad (r_0 < 30\ \mu m,\ h_{particle} = r_0) \qquad (S26)$$

$$r_{target} = \frac{3 \cdot h_{disk} \cdot IDR \cdot T_{si}}{Dn_{target} \cdot \rho \cdot h_{particle}} \qquad (r_0 \geq 30\ \mu m,\ h_{particle} = 30\ \mu m) \qquad (S27)$$

S.1.5. Dissolution/Permeation Balance: Derivation of Target Particle Size Equations based on k_{diss}/k_{perm} and Solubility

Equation (S28) serves as the starting point for the dissolution/permeation balance approach. The equation balances the initial (maximal) dissolution rate (DR_{max}) against the maximal permeation rate (PR_{max}).

$$DR_{max} = PR_{max} \qquad (S28)$$

The derivation of the dissolution rate term was presented previously (Equations (S4) and (S5) in Section S.1.1.) and leads to Equations (S29)–(S31).

$$k_{diss} \cdot \text{Dose} = PR_{max} \quad \text{(S29)}$$

$$\frac{3 \cdot D}{{r_0}^2} \cdot \frac{C_S}{\rho} \cdot \text{Dose} = PR_{max} \qquad (r_0 < 30\ \mu m,\ h_{particle} = r_0) \quad \text{(S30)}$$

$$\frac{3 \cdot D}{r_0 \cdot h_{particle}} \cdot \frac{C_S}{\rho} \cdot \text{Dose} = PR_{max} \qquad (r_0 \geq 30\ \mu m,\ h_{particle} = 30\ \mu m) \quad \text{(S31)}$$

The maximal permeation rate was estimated from the permeation rate constant (k_{perm}) and the maximum dissolved API amount in the effectively available 500 mL of intestinal fluid ($m_{dissolved}$) [7]. The k_{perm} (Equation (S33)) was derived from the Absorption Number (An, Equation (S32)), which represents the ratio of the radial absorption rate to axial convection rate [13].

$$An = \frac{T_{si}}{T_{perm}} = k_{perm} \cdot T_{si} = \frac{DF \cdot P_{eff}}{R_{si}} \cdot T_{si} \quad \text{(S32)}$$

$$k_{perm} = \frac{DF \cdot P_{eff}}{R_{si}} \quad \text{(S33)}$$

Multiplication of k_{perm} by the dissolved API amount results in the permeation rate according to Equation A34:

$$PR = k_{perm} \cdot m_{dissolved} = \frac{DF \cdot P_{eff}}{R_{si}} \cdot m_{dissolved} \quad \text{(S34)}$$

Incorporation of the permeation rate equation into Equations (S30) and (S31) leads to Equations (S35) and (S36).

$$\frac{3 \cdot D}{{r_0}^2} \cdot \frac{C_S}{\rho} \cdot \text{Dose} = \frac{DF \cdot P_{eff}}{R_{si}} \cdot m_{dissolved} \qquad (r_0 < 30\ \mu m,\ h_{particle} = r_0) \quad \text{(S35)}$$

$$\frac{3 \cdot D}{r_0 \cdot h_{particle}} \cdot \frac{C_S}{\rho} \cdot \text{Dose} = \frac{DF \cdot P_{eff}}{R_{si}} \cdot m_{dissolved} \qquad (r_0 \geq 30\ \mu m,\ h_{particle} = 30\ \mu m) \quad \text{(S36)}$$

Rearrangement for r_0 leads to the following r_{target} equations (Equations (S37) and (S38)):

$$r_{target} = \sqrt{\frac{3 \cdot D \cdot \text{Dose} \cdot R_{si} \cdot C_s}{DF \cdot P_{eff} \cdot \rho \cdot m_{dissolved}}} \qquad (r_0 < 30\ \mu m,\ h_{particle} = r_0) \quad \text{(S37)}$$

$$r_{target} = \frac{3 \cdot D \cdot \text{Dose} \cdot R_{si} \cdot C_s}{DF \cdot P_{eff} \cdot \rho \cdot m_{dissolved} \cdot h_{particle}} \qquad (r_0 \geq 30\ \mu m,\ h_{particle} = 30\ \mu m) \quad \text{(S38)}$$

S.1.6. Dissolution/Permeation Balance: Derivation of Target Particle Size Equations based on k_{diss}/k_{perm} and Intrinsic Dissolution Rate

The dissolution/permeation rate equilibrium can analogously be expressed using k_{diss} based on the IDR (Equations (S39) and (S40)):

$$\frac{3 \cdot h_{disk}}{r_0^2 \cdot \rho} \cdot IDR \cdot Dose = \frac{DF \cdot P_{eff}}{R_{si}} \cdot m_{dissolved} \quad (r_0 < 30\ \mu m,\ h_{particle} = r_0) \quad (S39)$$

$$\frac{3 \cdot h_{disk}}{r_0 \cdot h_{particle} \cdot \rho} \cdot IDR \cdot Dose = \frac{DF \cdot P_{eff}}{R_{si}} \cdot m_{dissolved} \quad (r_0 \geq 30\ \mu m,\ h_{particle} = 30\ \mu m) \quad (S40)$$

Rearrangement of these equations for r_0 yields the r_{target} equations (Equations (S41) and (S42)) presented in the main text as Equations (22) and (23):

$$r_{target} = \sqrt{\frac{3 \cdot h_{disk} \cdot Dose \cdot R_{si} \cdot IDR}{DF \cdot P_{eff} \cdot \rho \cdot m_{dissolved}}} \quad (r_0 < 30\ \mu m,\ h_{particle} = r_0) \quad (S41)$$

$$r_{target} = \frac{3 \cdot h_{disk} \cdot Dose \cdot R_{si} \cdot IDR}{DF \cdot P_{eff} \cdot \rho \cdot m_{dissolved} \cdot h_{particle}} \quad (r_0 \geq 30\ \mu m,\ h_{particle} = 30\ \mu m) \quad (S42)$$

S.2. Materials and Methods

S.2.1. Composition of FaSSIF V1

The composition of FaSSIF V1 is shown in Table S2 [14].

Table S2. Composition of FaSSIF V1.

Composition	FaSSIF V1
Sodium taurocholate (mM)	3
Lecithin (mM)	0.75
Sodium dihydrogen phosphate (mM)	28.65
Sodium chloride (mM)	105.85
Sodium hydroxide	10.5
Deionized water	q.s. 1 l
pH	6.5

S.2.2. Dissociation Constant (pK_a)

The pK_a values of voriconazole, lemborexant and istradefylline were determined with a high-throughput spectrometric screening assay (Fast UV method) using an automated titrator system with an incorporated UV-Vis spectrometer (SiriusT3, Pion Inc., Billerica, MA, USA). The experiments were performed at 25.0 ± 0.2 °C in a pH range covering pH 2 to 12. Samples (*n* = 1) were prepared by mixing an API stock solution in DMSO, Neutral Linear Buffer and water (with 0.15 M KCl) in the assay vial. Methanol was included as co-solvent, so that final concentrations ranged between 24.6 to 47.3% w/w. Strong acid or base was added to bring the pH to the target starting pH. The pK_a values were determined from three titrations with different water/methanol ratios and extrapolation of apparent pK_a values in the presence of co-solvents using the Yasuda-Shedlovsky extrapolation. Measured pK_a values are presented as means with standard deviation and were compared to literature values and predictions using the software ADMET Predictor

version 9 (Simulations Plus Inc., Lancaster, CA, USA) and ACD/Labs Release 2021.1.2 (Advanced Chemistry Development Inc., Toronto, Canada) with the molecular structure as input.

S.2.3. n-Octanol/Water Distribution Coefficient (logD$_{pH7.4}$)

The LogD$_{pH7.4}$ values of voriconazole, lemborexant and istradefylline were determined with a shake-flask screening method and HPLC analysis. Samples (*n* = 3) were prepared by addition of a 10 mM API stock solution in DMSO to an octanol saturated 0.01 M phosphate buffer at pH 7.4 to prepare a 1:10 dilution (ionic strength adjusted with 0.15 M NaCl). Buffer saturated octanol was added (1:1) to prepare partition systems, which were agitated on an electronic shake plate for 4 h at 25 ± 2°C. All samples were centrifuged, and aqueous and octanol layers were separately transferred to 96 well plates. Both layers of each replicate were analyzed with a standard HPLC gradient method (Table S3). A Waters Acquity H-Class Plus System consisting of a sample manager, a quaternary solvent manager and a photodiode array (PDA) detector was used. All HPLC data was collected and processed using the software Empower® version 3.0. Measured logD$_{pH7.4}$ values are presented as means with standard deviation and were compared to literature values and predictions using the software ADMET Predictor version 9 and ACD/Labs Release 2021.1.2 with the molecular structure as input.

Table S3. Standard HPLC method for the determination of logD$_{pH7.4}$ values.

<table>
<tr><th>System</th><th colspan="3">Waters Acquity H-Class Plus System</th></tr>
<tr><td>Column</td><td colspan="3">Reversed-phase Waters Acquity BEH C18, 50mm, 2.1mm 1.7 µm particle size</td></tr>
<tr><td>Mobile phase A</td><td colspan="3">0.1% Formic acid in water</td></tr>
<tr><td>Mobile phase B</td><td colspan="3">100% Acetonitrile</td></tr>
<tr><td>Run time</td><td colspan="3">2 min</td></tr>
<tr><td>Flow rate</td><td colspan="3">0.8 ml/min</td></tr>
<tr><td>Column temperature</td><td colspan="3">40 °C</td></tr>
<tr><td>Injection volume</td><td colspan="3">10 µL (aqueous layer), 0.1 µL (octanol layer)</td></tr>
<tr><td>Detection wavelength</td><td colspan="3">254 nm</td></tr>
<tr><td rowspan="6">Gradient time profile</td><td>Time [min]</td><td>%A</td><td>%B</td></tr>
<tr><td>Initial</td><td>90</td><td>10</td></tr>
<tr><td>1.0</td><td>10</td><td>90</td></tr>
<tr><td>1.3</td><td>10</td><td>90</td></tr>
<tr><td>1.4</td><td>90</td><td>10</td></tr>
<tr><td>2.0</td><td>90</td><td>10</td></tr>
</table>

S.2.4. rDCS Standard Investigations: Solubility Studies

Samples from the solubility studies were analyzed with UPLC-UV analysis. For voriconazole and lemborexant a Waters Acquity UPLC System consisting of a sample manager, a binary solvent manager, and a PDA detector was used (Waters Corporation, Milford, USA). For istradefylline a Waters Acquity UPLC H-Class System consisting of a sample manager, a quarternary solvent manager, and a PDA detector was used (Waters Corporation, Milford, MA, USA). The chromatograms were recorded and processed using the software Empower® version 3.0. Representative chromatograms for each API are shown in Figure S1 and the details of the UPLC methods are summarized in Tables S4–S7. The residual solids from the FaSSIF V1 solubility study were collected and examined using X-ray powder diffraction (XRPD) analysis to detect solution-mediated solid state changes. After 24 h, 1 to 5 mg of the residual solid was collected, placed on a zero-background holder and allowed to sit at room temperature for 2 h. The analysis was performed on a PANalytical X'PertPRO MPD diffractometer equipped with a Cu LFF X-ray tube (Philips, Amsterdam, The Netherlands) from 3° to 50° 2θ and a step size of 0.02°. Cu Kα radiation with a wavelength of 1.54 Å was used at a generator voltage of 45 kV and a generator amperage of 40 mA. XRPD patterns of the residual solids were compared to the starting material and literature profiles. A differential scanning calorimetry (DSC) analysis of the input materials was additionally performed. For this, 2 to 3 mg of the material were weighed into a standard aluminum DSC pan. The pan was closed with an aluminum lid and heated from 25°C to 180 °C (voriconazole), 210 °C (lemborexant) or 250 °C (istradefylline) at 10 °C/min in a TA Instruments DSC 2500 (TA Instruments, New Castle, DE, USA) equipped with a refrigerated cooling system. The DSC thermograms were compared to literature data.

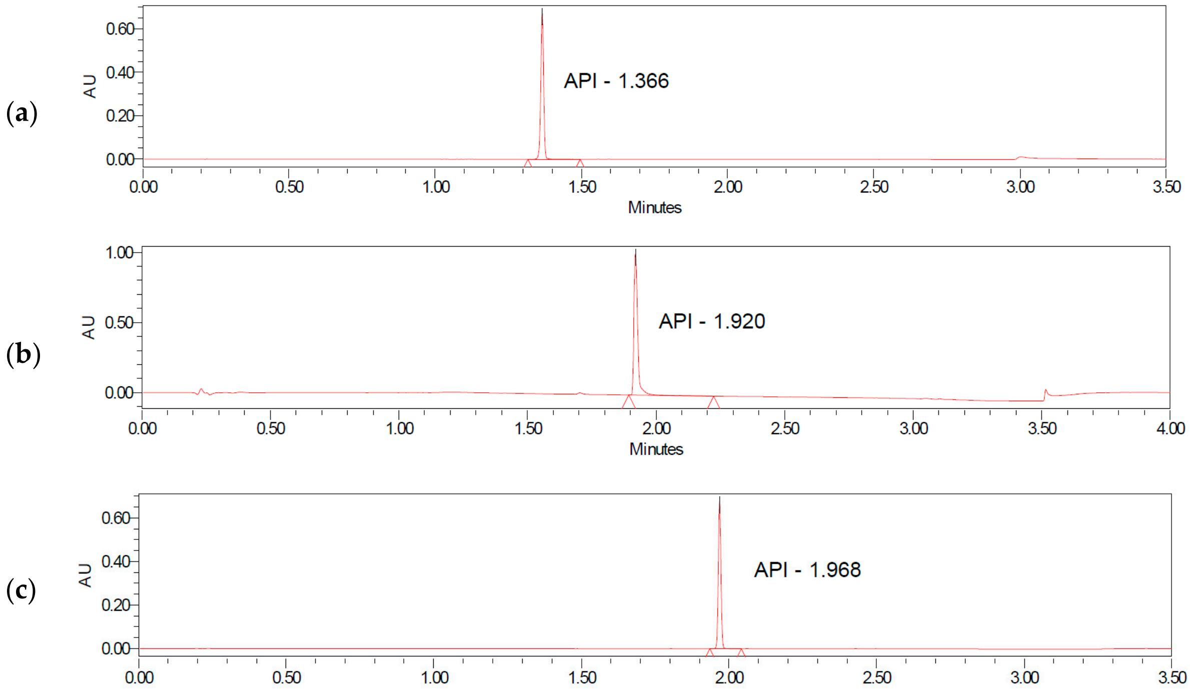

Figure S1. Exemplary chromatograms of the highest standards from the calibration curve. (**a**) voriconazole, (**b**) lemborexant, (**c**) istradefylline.

Table S4. Details of the UPLC methods for quantification of API concentrations in solubility study samples.

API	Voriconazole	Lemborexant	Istradefylline
System	Waters Acquity UPLC System	Waters Acquity UPLC System	Waters Acquity UPLC H-Class System
Column	Reversed-phase Waters Acquity BEH C18, 50mm, 2.1mm 1.7 µm particle size		
Mobile phase A	0.1 % TFA in Milli-Q water		
Mobile phase B	100% Acetonitrile		
Gradient time profile	A gradient mobile phase A and B was used for separation. The details of the gradient time profiles can be found in Tables S5–S7.		
Run time	3.5 min	4 min	3.5 min
Flow rate	0.6 ml/min	0.6 ml/min	0.6 ml/min
Column temperature	55 °C	55 °C	55 °C
Injection volume	2 µl	2 µl	4 µl
Detection wavelength	256 nm	229 nm	361 nm
Retention time	1.37 min	1.92 min	1.97 min
Linear range	1-100 µg/ml	1-100 µg/ml	0.1-10 µg/ml
Coeff. of correlation (R^2)	0.999985	0.999998	0.999996
Recovery of control	99.89 %	99.25 %	99.97 %
Limit of quantification	< 1 µg/ml	< 0.05 µg/ml	< 0.1 µg/ml
Repeatability (% RSD of highest standard)	0.07 %	0.04 %	0.04 %

Table S5. Gradient of the UPLC method for analysis of voriconazole.

Time (min)	%A	%B
Initial	90	10
0.3	90	10
2.3	0	100
2.6	0	100
2.7	90	10
3.5	90	10

Table S6. Gradient of the UPLC method for analysis of lemborexant.

Time (min)	%A	%B
Initial	85	15
0.8	85	15
2.8	0	100
3.1	0	100
3.2	85	15
4.0	85	15

Table S7. Gradient of the UPLC method for analysis of istradefylline.

Time (min)	%A	%B
Initial	85	15
0.3	85	15
2.3	0	100
2.6	0	100
2.7	85	15
3.5	85	15

S.2.5. rDCS Standard Investigations: Caco-2 permeability studies

Caco-2 cells were obtained from the American Type Culture Collection (ATCC, Manassas, VA, USA) and used between passage numbers 40 to 60. The cells were seeded onto Millipore Multiscreen Transwell plates at a density of 1 x 10^5 cells/cm^2 and cultured in Dulbecco's Modified Eagle's Medium (DMEM). The medium was changed every two or three days. Cell culture was carried out at 37 °C in an atmosphere of 5 % CO_2 with a relative humidity of 95 %. The permeability assay was performed on day 20.

In vitro permeability was assessed with a high-throughput Caco-2 permeability assay. The assay was prepared by rinsing apical and basolateral monolayer surfaces twice with Hanks Balanced Salt Solution (HBSS) at the desired pH and 37 °C. Subsequently, cells in the apical and basolateral compartments were incubated with HBSS at the desired pH for 40 min. A pH gradient with pH 6.5 in the apical compartment and pH 7.4 in the basolateral compartment was applied. Dosing solutions containing the API (10 µM) were prepared by diluting a concentrated API solution in DMSO with HBSS. The final DMSO concentration was ≤ 1% v/v. Lucifer yellow (100 µM) was included in the dosing solution as fluorescent integrity marker. For the assessment of the permeability from the apical to basolateral side (A⇒B), HBSS in the apical compartment was replaced with the dosing solution. The apical inserts were placed into a companion plate containing fresh buffer with 1% v/v DMSO. For the assessment of the permeability from the basolateral to

apical side (B⟹A), HBSS was removed from the companion plate and replaced with the solution of drug in HBSS. Fresh HBSS was added to the apical insert, which was then placed into the companion plate. Experiments were performed in absence and presence of the P-glycoprotein (P-gp) inhibitor elacridar (10 μM) to study transporter-mediated efflux effects. In those experiments, elacridar was included in both compartments during the equilibration time and assay. The starting concentration (C_0) was confirmed by sampling from the dosing solution immediately before dosing into the cell plates. After incubation of 120 minutes, the apical inserts and companion plates were separated for sampling from both compartments. Samples were diluted for analysis via liquid chromatography coupled with tandem mass spectrometry (LC-MS/MS). The LC system consisted of a column manager, binary solvent manager, autosampler and a Xevo TQ MS Triple Quadrupole Mass Spectrometer (Waters Ltd., Herts, UK). Details of the analytical standard method are presented in Table S8. Measurements were performed in duplicate and reference compounds of known permeability (atenolol and antipyrine) were included as quality controls on each plate. Propranolol was included as an additional test compound for data analysis purposes.

The apparent permeability coefficients (P_{app}) were calculated according to Equation (S43):

$$P_{app} = \left(\frac{dQ/dt}{C_0 \cdot A}\right) \tag{S43}$$

where dQ/dt is permeation rate of the API across the cell monolayer, C_0 is starting concentration of the API in the dosing solution and A is surface area of the cell monolayer [15]. P_{app} values were averaged. To estimate whether P-gp mediated efflux was involved in the transport of the APIs, efflux ratios (ER) were calculated from the mean A⟹B and B⟹A P_{app} values with and without inhibitor (Equation (S44)). An ER of ≥ 2 in cells expressing P-gp suggests that an investigational API is an in vitro P-gp substrate [16]. The mass balance (recovery) was calculated from the sum of the API recovered from the acceptor and donor compartments at the end of the experiment, divided by the initial donor amount [15]. The lucifer yellow permeability was monitored during the experiment using fluorimetric analysis. The pre-defined lucifer yellow P_{app} acceptance threshold was 0.5 x 10^{-6} cm/s.

$$ER = \frac{P_{app(B \Rightarrow A)}}{P_{app(A \Rightarrow B)}} \tag{S44}$$

Averaged P_{app}(A⟹B) values without inhibitor were converted to effective human *in vivo* permeability (P_{eff}) values using Simcyp Version 21 software (Certara UK Ltd., Sheffield, UK). The built-in literature P_{app}-P_{eff} correlation assuming a pH gradient (pH 6.5 to 7.4) and passive API permeation with atenolol and

propranolol as calibrator substances was used [17]. Estimated P_{eff} values were used as input for the rDCS and r_{target} calculations.

Table S8. Details of the LC-MS/MS method for quantification of API concentrations in Caco-2 permeability study samples.

<table>
<tr><td>Column</td><td colspan="3">Acquity™ HSS T3 (1.8 µm) 2.1 x 30 mm (Waters Ltd, Herts, UK) fitted with SecurityGuard™ ULTRA Fully Porous Polar C18 cartridge (Phenomenex, Cheshire, UK)</td></tr>
<tr><td>Mobile phase A</td><td colspan="3">10 mM ammonium formate + 0.1 % v/v formic acid in water</td></tr>
<tr><td>Mobile phase B</td><td colspan="3">100% Methanol</td></tr>
<tr><td rowspan="7">Gradient time profile</td><td>Time (min)</td><td>%A</td><td>%B</td></tr>
<tr><td>Initial</td><td>100</td><td>0</td></tr>
<tr><td>0.03</td><td>100</td><td>0</td></tr>
<tr><td>0.60</td><td>5</td><td>95</td></tr>
<tr><td>0.65</td><td>5</td><td>95</td></tr>
<tr><td>0.80</td><td>100</td><td>0</td></tr>
<tr><td>0.90</td><td>100</td><td>0</td></tr>
<tr><td>Run time</td><td colspan="3">0.9 min</td></tr>
<tr><td>Flow rate</td><td colspan="3">1 mL/min</td></tr>
<tr><td>Column temperature</td><td colspan="3">40 °C</td></tr>
<tr><td>Injection volume</td><td colspan="3">12 µL</td></tr>
</table>

S.2.6. rDCS Customized Investigations: Intrinsic dissolution rate

The concentration-time curves for IDR determination were recorded with in situ UV-spectroscopy using fiber optic dip probes. Each probe was calibrated individually before the experiment was started. Pre-heated FaSSIF V1 served as starting point of the calibration curves and subsequently five different API concentrations in FaSSIF V1 were produced by spiking a concentrated stock solution of the API in DMSO into the medium. The buffer was stirred at 100 rpm and spectra were recorded approximately 1 minute after addition of the stock solution. The standard curve concentrations were chosen to capture the range of 0 to 10 % (voriconazole and lemborexant) or 0 to 100 % (istradefylline) of the API solubility in FaSSIF V1. Second derivatives of the UV spectra were used to establish the calibration curves. Multiple wavelength ranges were evaluated, and calibration curves were accepted for further investigation when linear regression resulted in a coefficient of correlation (R^2) ≥ 0.99. The final range for voriconazole and lemborexant was selected based on recovery measurements with control solutions containing a known API concentration in FaSSIF V1. For istradefylline, recoveries were calculated based on samples taken and analyzed with UPLC-UV at the end of the dissolution experiment. The wavelength ranges with the best R^2 and % recovery values were selected for in-line concentration analysis. Parameters and results of the control measurements are summarized in Table S9.

Table S9. Parameters of the in-line UV methods for IDR determination.

Parameter	Voriconazole	Lemborexant	Istradefylline
Mirror path length [mm]	1	10	20
Wavelength range [nm]	275-290	280-300	350-370
Conc. range (standard curve) [µg/mL]	0-241	0-3.67	0-3.85
Coefficient of correlation (R^2)	0.9981-0.9996	0.9999-1.000	0.9999-1.000
Recovery of control solution [%]	98.6 ± 2.0	100.0 ± 2.1	95.1 ± 0.6

XRPD analysis of the disks before and after dissolution was performed. For the pre-experiment examination, disks were prepared and carefully crushed using a mortar and pestle. Care was taken to exert as little force as possible. The material was transferred to a zero-background holder for analysis. For the post-experiment examination, the disks were recovered after the dissolution experiment. Disks were allowed to sit at room temperature for approximately 2 h and subsequently crushed and transferred to zero-background holders for analysis. The lemborexant disk (pre-experiment) was additionally analyzed by DSC because small differences in the XRPD patterns of the disks and powder starting material were observed. The uniformity of the disk surfaces was examined microscopically before and after the

experiments using a ZEISS Axio Vert.A1 Inverted Microscope for Advanced Routine (Carl Zeiss Microscopy GmbH, Jena, Germany).

S.2.7. rDCS Calculations: Customized Investigations

Table S10 shows the predicted aqueous diffusion coefficients and API densities used for the calculation of r_{target} in the rDCS customized investigations.

Table S10. Predicted aqueous diffusion coefficients and API densities for r_{target} calculations.

API	Aqueous diffusion coefficient [1] [x 10^{-6} cm^2/s]	API density [2] [g/cm^3]
**Voriconazole**	7.46	1.43
**Lemborexant**	6.53	1.35
**Istradefylline**	6.53	1.24

[1] Predicted with ADMET Predictor version 9.
[2] Predicted with ACD/Labs Release 2021.1.2.

S.3. Results

S.3.1. Dissociation Constant (pK_a)

Experimental, in silico predicted and literature pK_a values for voriconazole, lemborexant and istradefylline are summarized in Table S11.

Table S11. Experimental, literature and in silico predicted pK_a values of voriconazole, lemborexant and istradefylline.

Method for pK_a determination	Voriconazole	Lemborexant	Istradefylline
Fast UV	- [1]	2.18 ± 0.05 [2]	- [1]
Literature pK_a	1.76 [3] [18]	< 3.50 [19]	0.78 [3] [20, 21]
ADMET Predictor pK_a	3.14	1.57	2.94
		3.45	
ACD/Labs pK_a (Classic)	2.30	1.80	0.50
		2.50	
ACD/Labs pK_a (GALAS)	1.30	1.70	- [4]
		4.00	

[1] Evidence of low pK_a outside the calibration limit (titration pH 2 to 12).
[2] Low absorbance (< 0.30 abs units) associated with the pK_a, value should be treated with caution.
[3] Method of determination not disclosed.
[4] No ionization constant predicted.

The pK_a of lemborexant, determined with the Fast UV method, was 2.18 ± 0.05, noting that the value was associated with a low absorbance in the assay. Comparative literature values were < 3.5 by capillary electrophoresis [19], while in silico predictions identified two pK_a values: a low pK_a of 1.50 to 1.70 for the pyridine structure and a higher pK_a of 2.50 to 4.00 for the pyrimidine group.

The experimental pK_a values of voriconazole and istradefylline lay outside the standard titration range of pH 2 to 12. In both cases a pK_a below the lower calibration limit of the Fast UV method was detected, but not reported. For voriconazole, a basic pK_a of 1.76 associated with the triazole moiety was reported in the literature [18], while in silico predictions for voriconazole varied from 1.30 to 3.14. For istradefylline, a basic pK_a of 0.78 was reported in the literature [20], while the in silico predictions were 0.50 or 2.94, depending on the algorithm applied.

S.3.2. n-Octanol/Water Distribution Coefficient ($logD_{pH7.4}$)

Experimental, in silico predicted and literature $logD_{pH7.4}$ or logP values for the three APIs are summarized in Table S12. Based on the pK_a values, all compounds were unionized at pH 7.4, therefore the $logD_{pH7.4}$ and logP values were identical. The literature $logD_{pH7.4}$ values differed from measured values by less than 10% (voriconazole), less than 25% (lemborexant) and less than 25% (istradefylline). In silico predictions differed from the measured values by less than 50% (voriconazole), less than 25% (lemborexant) and less than 35% (istradefylline).

Table S12. Experimental, literature and in silico predicted $logD_{pH7.4}$/logP [1] of voriconazole, lemborexant and istradefylline.

Method for $logD_{pH7.4}$/logP determination	Voriconazole	Lemborexant	Istradefylline
Shake-flask $logD_{pH7.4}$	1.66 ± 0.03	3.04 ± 0.05	2.96 ± 0.03
Literature logP [2]	1.80 [18]	3.70 [19]	3.5-3.6 [21]
ADMET Predictor MlogP	2.46	3.00	1.99
ADMET Predictor S+logP	2.13	3.76	2.85
ADMET Predictor S+$logD_{pH7.4}$	2.13	3.76	2.85
ACD/Labs $logD_{pH7.4}$	1.39	3.29	2.54
ACD/Labs logP (Classic)	0.93	3.16	2.84
ACD/Labs logP (GALAS)	1.63	3.42	2.41
ACD/Labs logP (Consensus)	1.39	3.29	2.54

[1] As none of the three compounds are ionized at pH 7.4, the logP and $logD_{pH7.4}$ should be identical.
[2] Method of determination not disclosed.

S.3.3. rDCS Standard Investigations: Solubility Studies

Overlays of the XRPD patterns of the starting materials and residual solids from the solubility studies are shown in Figures S2–S4. Figures S5–S7 show the DSC thermograms of the starting materials.

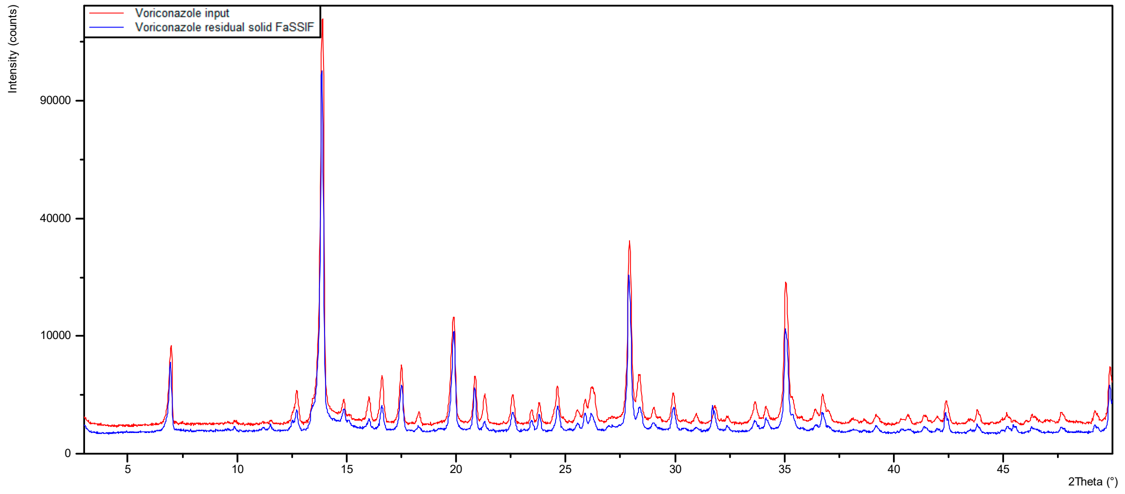

Figure S2. Overlay of the XRPD patterns of the voriconazole starting material and residual solid from the solubility study. The red pattern is shifted upwards for better comparability of the curves.

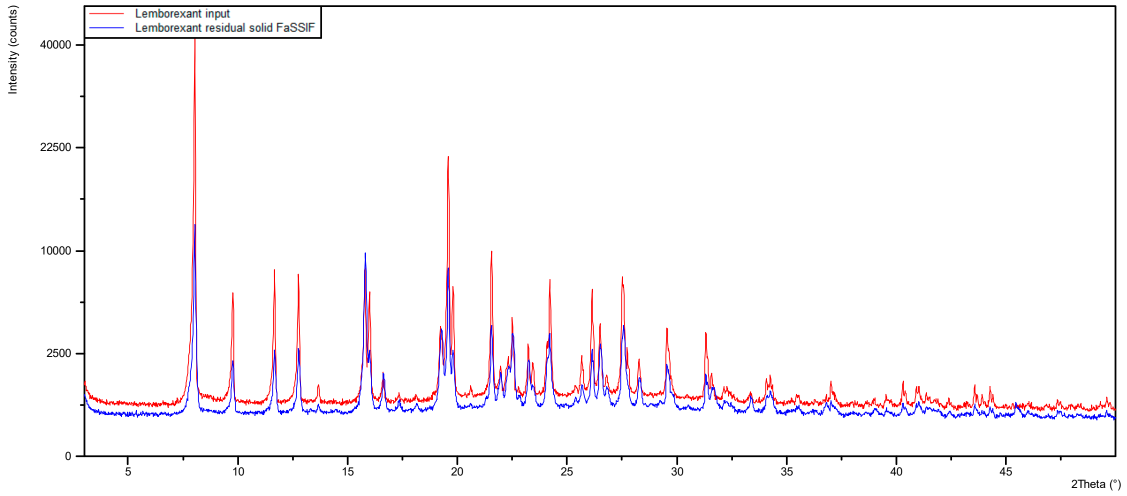

Figure S3. Overlay of the XRPD patterns of the lemborexant starting material and residual solid from the solubility study. The red pattern is shifted upwards for better comparability of the curves.

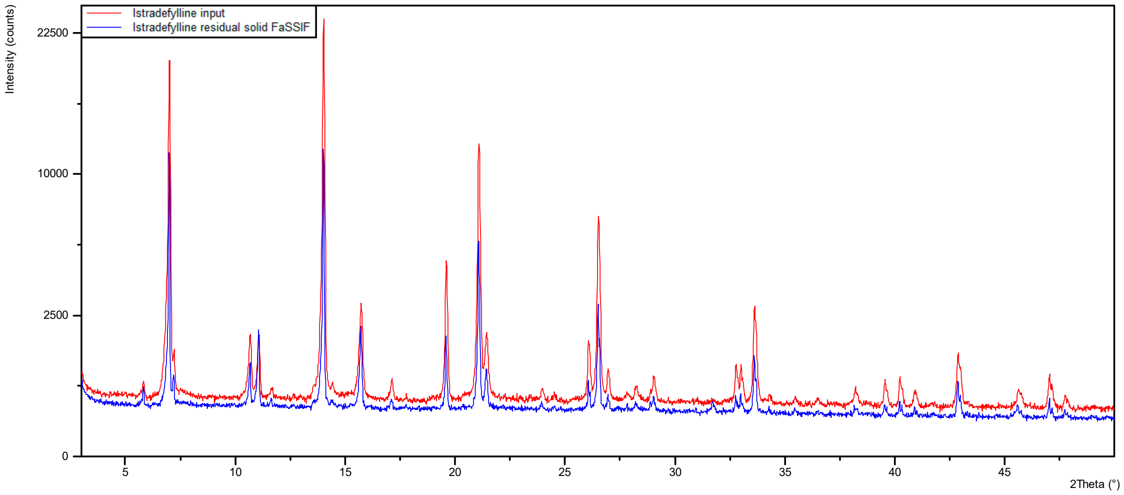

Figure S4. Overlay of the XRPD patterns of the istradefylline starting material and residual solid from the solubility study. The red pattern is shifted upwards for better comparability of the curves.

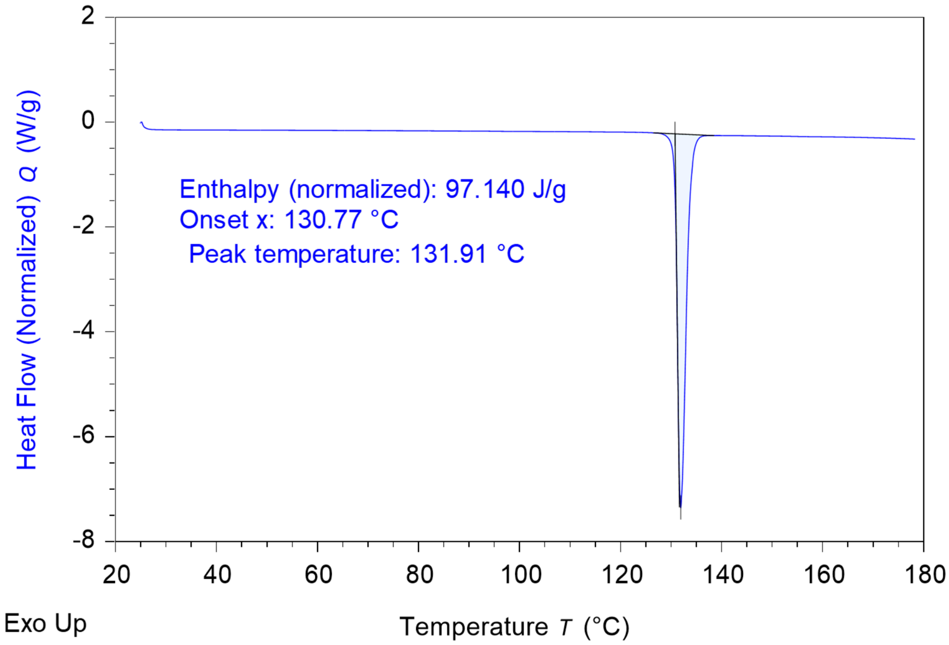

Figure S5. DSC thermogram of voriconazole starting material.

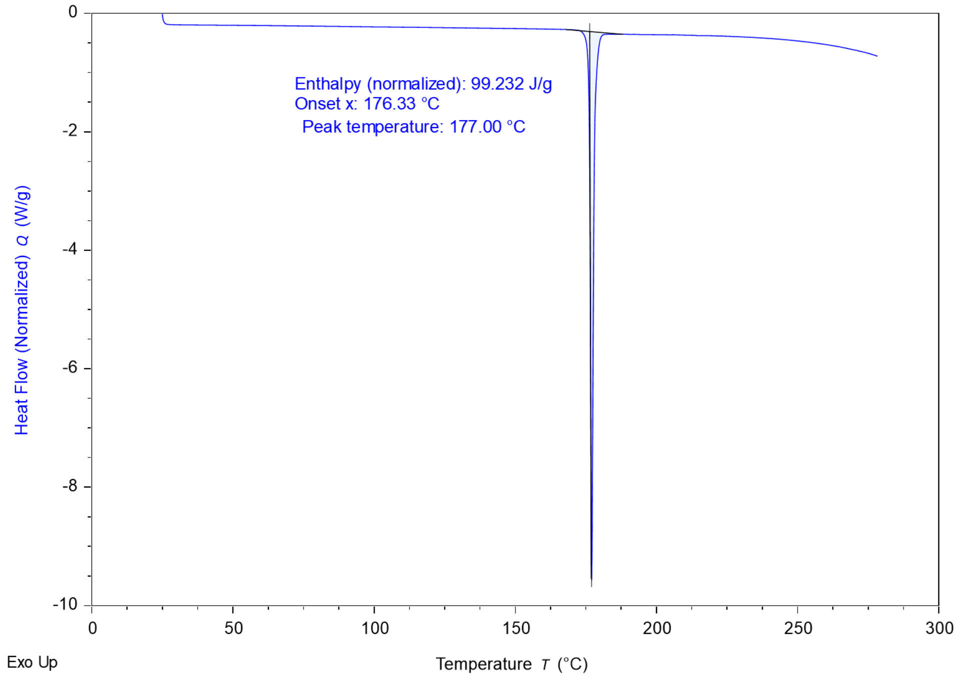

Figure S6. DSC thermogram of lemborexant starting material.

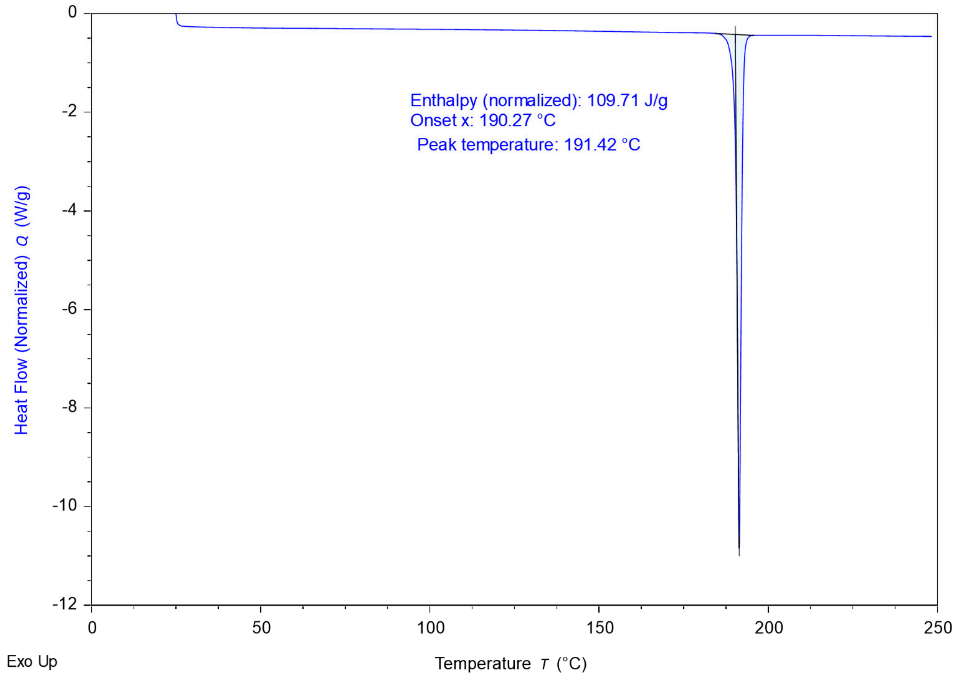

Figure S7. DSC thermogram of istradefylline starting material.

S.3.4. rDCS Customized Investigations: Intrinsic Dissolution Rate

Figures S8–**Figure** S10 show the disk and powder dissolution curves of the three APIs that were used to calculate the IDRs.

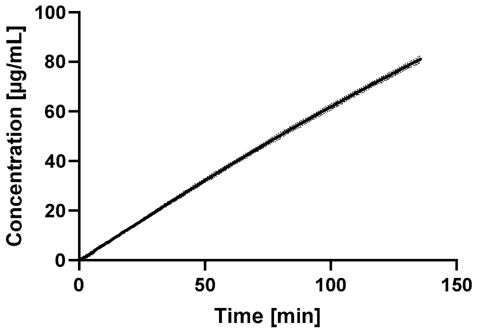

Figure S8. Voriconazole disk dissolution curve in FaSSIF V1 (mean ± SD).

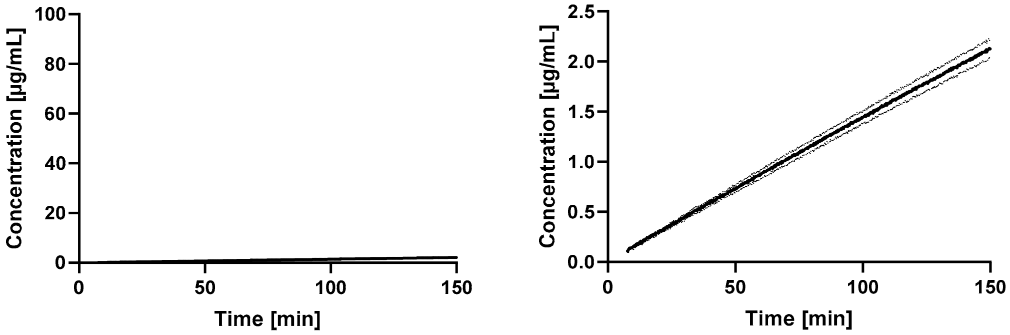

Figure S9. Lemborexant disk dissolution curve in FaSSIF V1 with different scaling of the y-axis (mean ± SD).

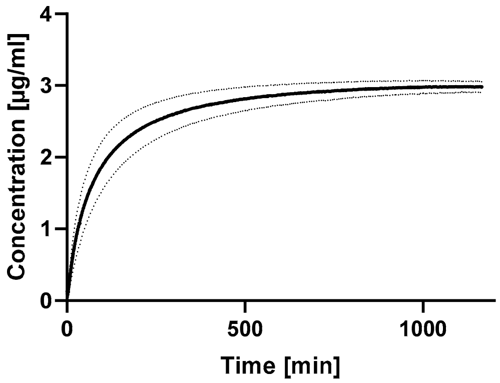

Figure S10. Istradefylline powder dissolution curve in FaSSIF V1 (mean ± SD).

Overlays of the XRPD patterns comparing the materials used for disk compression and the disks pre- and post-dissolution are shown in Figures S11–S13. Figures S14 and S15 show the DSC thermograms of the lemborexant and istradefylline disks pre-experiment compared to the input material. The XRPD patterns of the voriconazole starting material and disks (Figure S11) were identical and assigned to crystalline form B [22]. The XRPD patterns of the lemborexant starting material and disks (Figure S12) were similar with small differences between 13 to 15° and 20 to 30° 2θ. However, the DSC analysis of the disk (Figure S14) confirmed crystalline Form CS2. Small differences in the XRPD patterns can be explained by different morphologies of the samples. For particles of the same material but different morphology, the relative intensity of peaks can change. This phenomenon is referred to as preferred orientation [23]. In this study, the lemborexant input material was needle shaped, but the particle morphology changed due to compression and subsequent crushing. This caused small differences in the XRPD patterns, which are not reflective of a solid state change. XRPD and DSC analysis showed that compression of istradefylline into disks led to a solid state change (Figures S13 and S15). Hence, the disk IDR could not be measured and was estimated by powder dissolution experiments. The solid state of istradefylline in the powder dissolution experiment was inferred from the solubility study because excess solid could not be recovered from the powder IDR study.

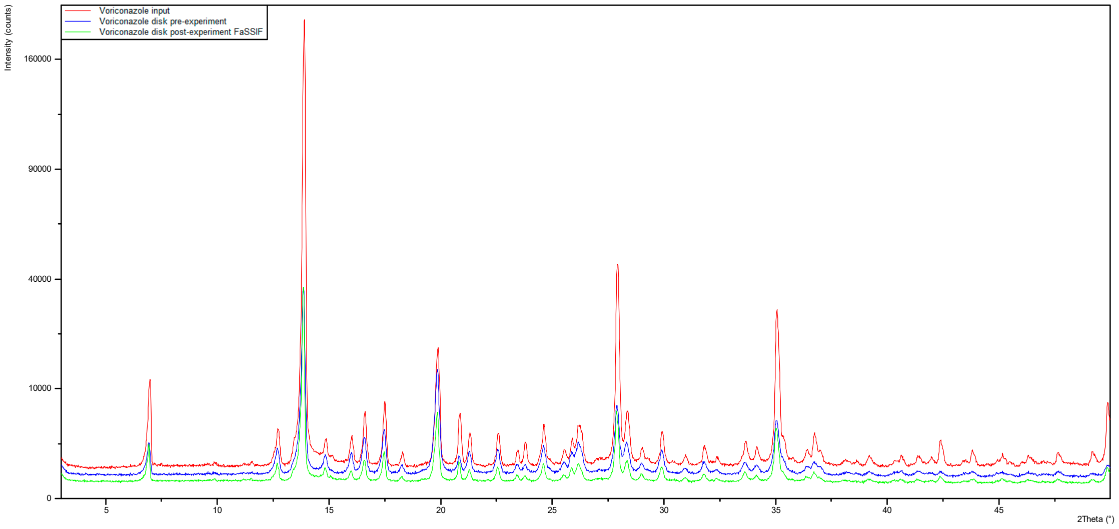

Figure S11. XRPD patterns of voriconazole starting material and voriconazole disks pre-/post-experiment. The red and blue patterns are shifted upwards for better comparability of the curves.

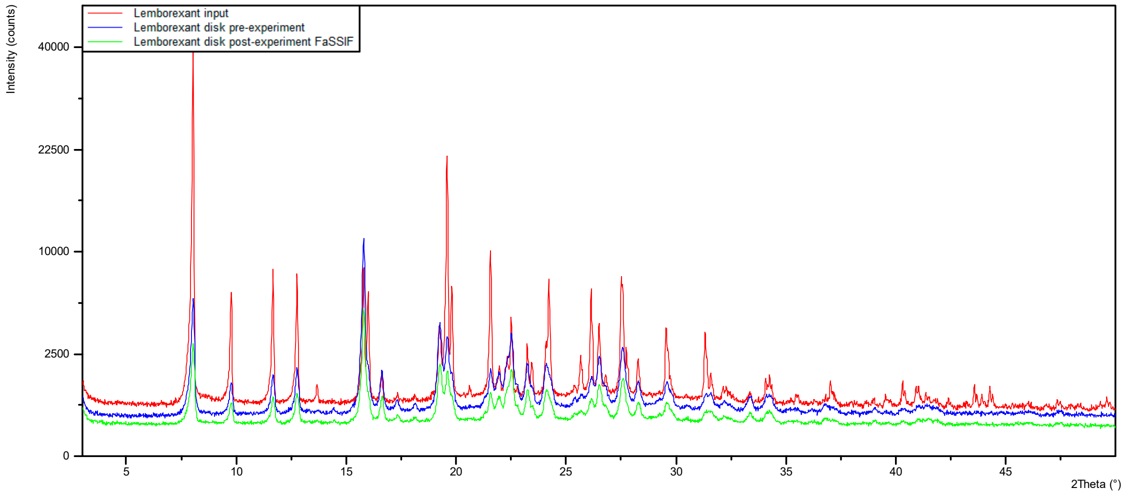

Figure S12. XRPD patterns of lemborexant starting material and disks pre-/post-experiment. The red and blue patterns are shifted upwards for better comparability of the curves.

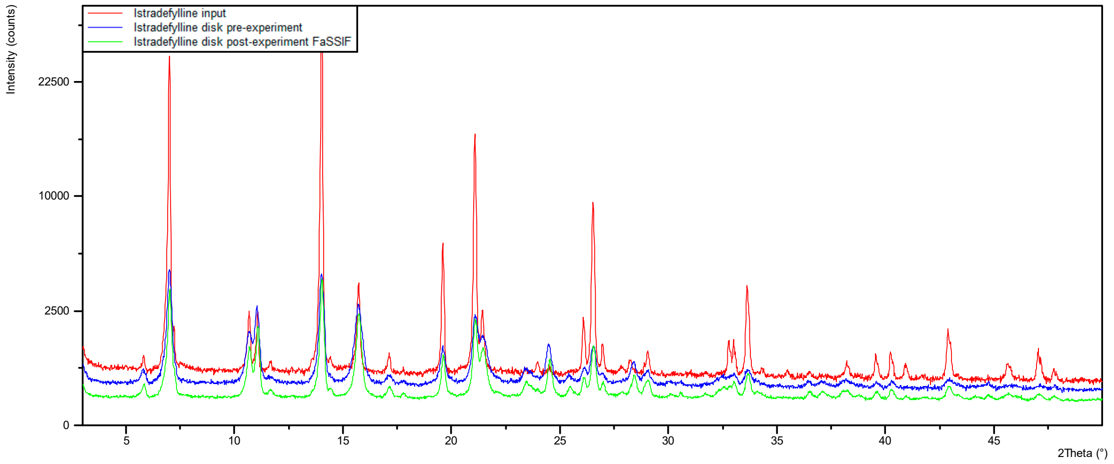

Figure S13. XRPD patterns of istradefylline starting material and disks pre-/post-dissolution (IDR was experimentally determined using the powder dissolution method). The red and blue patterns are shifted upwards for better comparability of the curves.

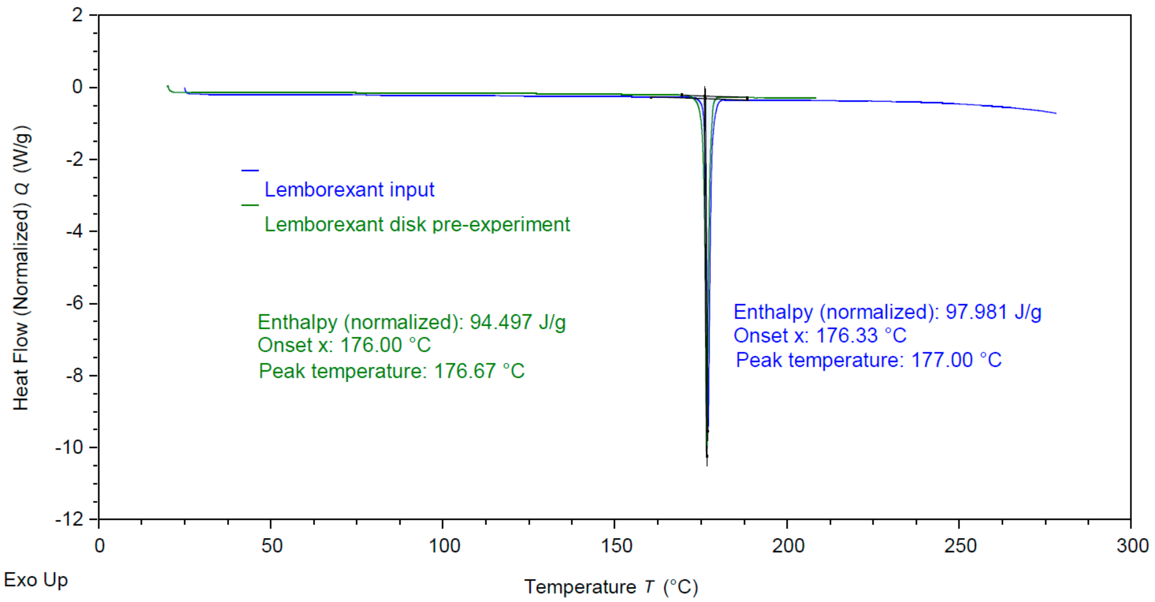

Figure S14. DSC thermogram of lemborexant starting material and lemborexant disk pre-experiment.

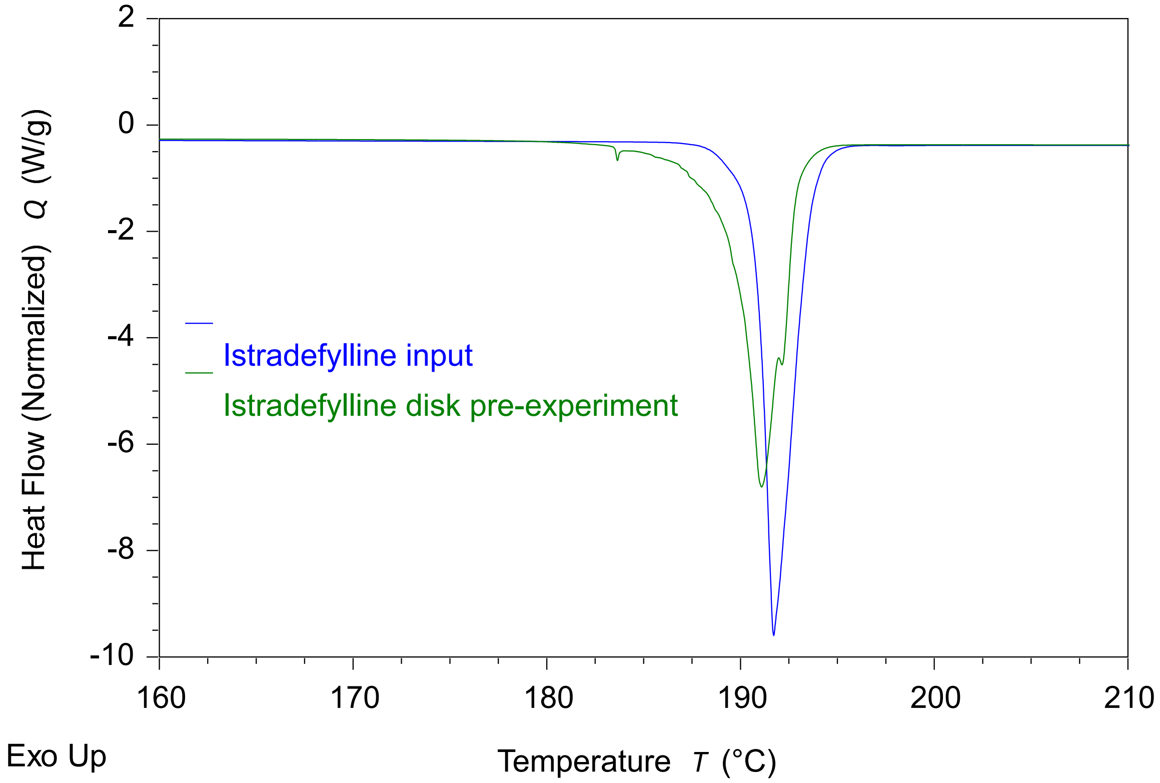

Figure S15. DSC thermogram of istradefylline starting material and istradefylline disk pre-experiment.

Figures S16 and S17 show the results of the optical and microscopical inspection of the disks, and Figure S18 shows the morphology of the istradefylline input material.

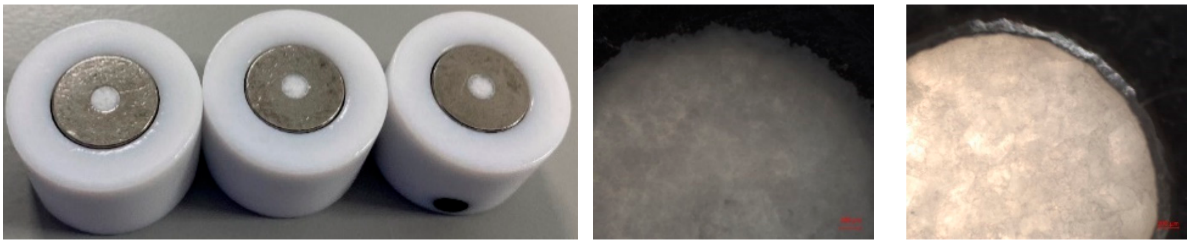

(**a**) (**b**) (**c**)

Figure S16. Voriconazole disks, (**a**) pre-experiment, (**b**) pre-experiment (microscopically), (**c**) post-experiment (microscopically).

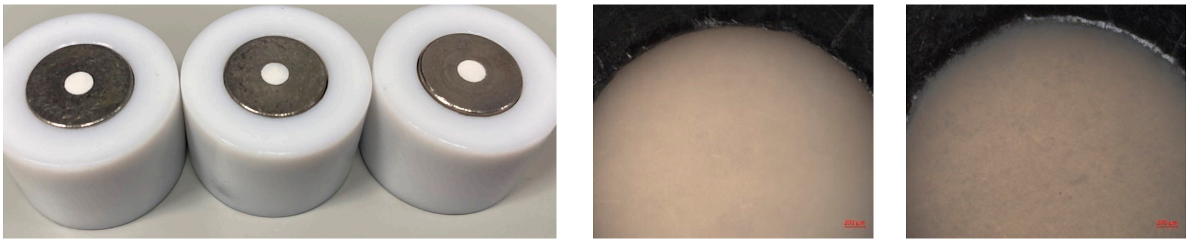

(**a**) (**b**) (**c**)

Figure S17. Lemborexant disks, (**a**) pre-experiment, (**b**) pre-experiment (microscopically), (**c**) post-experiment (microscopically).

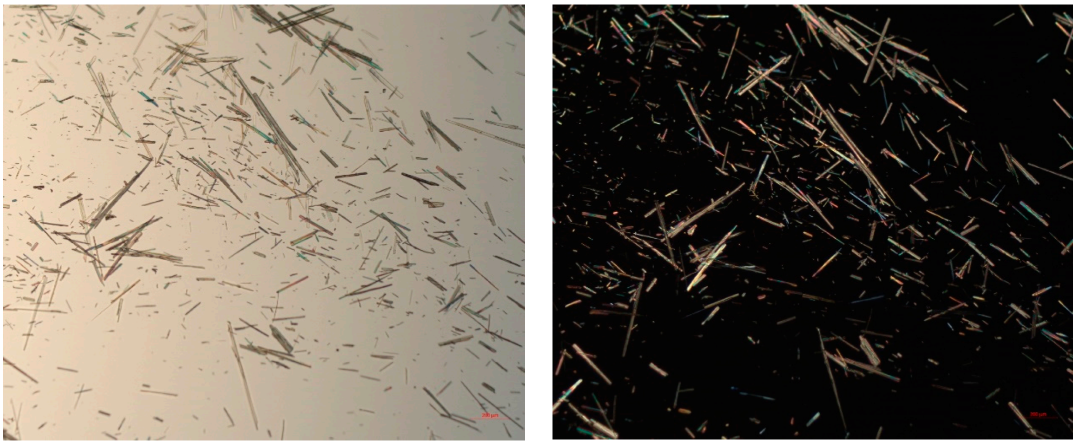

(**a**) (**b**)

Figure S18. Needle-shaped istradefylline input material under (**a**) normal light, and (**b**) polarized light.

References

1. Nernst, W. Theorie der Reaktionsgeschwindigkeit in heterogenen Systemen. *Z Phys Chem* **1904**, *47*, 52-55.
2. Brunner, E. Reaktionsgeschwindigkeit in heterogenen Systemen. *Z Phys Chem* **1904**, *43*, 56-102.
3. Noyes, A.A.; Whitney, W.R. The rate of solution of solid substances in their own solutions. *J Am Chem Soc* **1897**, *19*, 930-934.
4. Dokoumetzidis, A.; Macheras, P. A century of dissolution research: From Noyes and Whitney to the Biopharmaceutics Classification System. *Int J Pharm* **2006**, *321*, 1-11.
5. Dressman, J.B.; Fleisher, D. Mixing-tank model for predicting dissolution rate control or oral absorption. *J Pharm Sci* **1986**, *75*, 109-116.
6. Higuchi, W.I.; Hiestand, E.N. Dissolution rates of finely divided drug powders I. Effect of a distribution of particle sizes in a diffusion-controlled process. *J Pharm Sci* **1963**, *52*, 67-71.
7. Sugano, K. Biopharmaceutics Modelling and Simulations: Theory, Practice, Methods, and Applications, 1st ed.; John Wiley & Sons, Inc.: Hoboken, NJ, USA, 2012; pp. 1–325.
8. Hintz, R.J.; Johnson, K.C. The effect of particle size distribution on dissolution rate and oral absorption. *Int J Pharm* **1989**, *51*, 9-17.
9. Okazaki, A.; Mano, T.; Sugano, K. Theoretical dissolution model of poly-disperse drug particles in biorelevant media. *J Pharm Sci* **2008**, *97*, 1843-1852.
10. Arav, Y.; Bercovier, M.; Parnas, H. Selecting the particle size distribution for drugs with low water solubility – mathematical model. *Drug Dev Ind Pharm* **2012**, *38*, 940-951.
11. Levich, V.G. *Physicochemical Hydrodynamics*, 2nd ed.; Prentice Hall, Inc.: Englewood Cliffs, New Jersey, USA, 1962; pp. 39-72
12. Rosenberger, J.; Butler, J.; Dressman, J. A Refined Developability Classification System. *J Pharm Sci* **2018**, *107*, 2020-2032.
13. Oh, D.M.; Curl, R.L.; Amidon, G.L. Estimating the Fraction Dose Absorbed from Suspensions of Poorly Soluble Compounds in Humans: A Mathematical Model. *Pharm Res* **1993**, *10*, 264-270.
14. Biorelevant.com. Biorelevant Media Prep Tool 2022. Available online: https://biorelevant.com/?filters=product_type--biorelevant%20media#media_prep_tool_tab (accessed on 25 May 2022).
15. Hubatsch, I.; Ragnarsson, E.G.; Artursson, P. Determination of drug permeability and prediction of drug absorption in Caco-2 monolayers. *Nat Protoc* **2007**, *2*, 2111-2119.
16. Food and Drug Administration. *In Vitro Metabolism and Transporter Mediated Drug-Drug Interaction Studies Guidance for Industry*; U.S. Food and Drug Administration: Silver Spring, MD, USA, 2017. Available online: https://www.fda.gov/media/108130/download (accessed on 05 July 2023).
17. Sun, D.; Lennernäs, H.; Welage, L.S.; Barnett, J.L.; Landowski, C.P.; Foster, D.; Fleisher, D.; Lee, K.; Amidon, G.L. Comparison of Human Duodenum and Caco-2 Gene Expression Profiles for 12,000 Gene Sequences Tags and Correlation with Permeability of 26 Drugs. *Pharm Res* **200**, *19*, 1400-1416.
18. Damle, B.; Varma, M.V.; Wood, N. Pharmacokinetics of Voriconazole Administered Concomitantly with Fluconazole and Population-Based Simulation for Sequential Use. *Antimicrob Agents Chemother* **2011**, *55*, 5172-5177.
19. Ueno, T.; Miyajima, Y.; Landry, I.; Lalovic, B.; Schuck, E. Physiologically-based pharmacokinetic modeling to predict drug interactions of lemborexant with CYP3A inhibitors. *CPT Pharmacometrics Syst. Pharmacol* **2021**, *10*, 455-466.
20. Kyowa Kirin Inc. *Highlights of Prescribing information, NOURIANZ® (Istradefylline) Tablets, for Oral Use*; Kyowa Kirin Inc.: Bedminster, NJ, USA, 2020. Available online: https://www.nourianzhcp.com/ (accessed on 05 July 2023).
21. Food and Drug Administration. *Product Quality Review(s), Nourianz® (Istradefylline) Tablets. Application Number: 022075Orig1s000*; U.S. Food and Drug Administration: Silver Spring, MD, USA, 2019. Available online: https://www.accessdata.fda.gov/drugsatfda_docs/nda/2019/022075Orig1s000TOC.cfm (accessed on 05 July 2023).
22. Sundaram, V.; Uppala, V.B.R.U.; Akundi, S.P.; Muvva, V.; Chitta, V.; Donthula, A; Kharkar, M.R.; Devarakonda, S.N.; Peddireddy, S.R. Process for preparing voriconazole. Patent WO 2006/065726 A2, 22 June 2006.

23. Holder, C.F.; Schaak, R.E. Tutorial on Powder X-ray Diffraction for Characterizing Nanoscale Materials. *ACS Nano* **2019**, *13*, 7359-7365.
